# Flow cytometry-based diagnostic approach for inborn errors of immunity: experience from Algeria

**DOI:** 10.3389/fimmu.2024.1402038

**Published:** 2024-07-12

**Authors:** Azzeddine Tahiat, Reda Belbouab, Abdelghani Yagoubi, Saliha Hakem, Faiza Fernini, Malika Keddari, Hayet Belhadj, Souad Touri, Samira Aggoune, Jennifer Stoddard, Julie Niemela, Farida Zerifi, Souhila Melzi, Rawda Aboura, Amina Saad-Djaballah, Yacine Ferhani, Abdalbasset Ketfi, Hassen Messaoudi, Tahar Bencharif Madani, Zouleikha Benhacine, Abdelhak Dehimi, Kamelia Okka, Fairouz Amroune, Meriem Fellahi, Chafa Bendahmane, Radia Khoulani, Asma Oukil, Asma Soufane, Imene Bourelaf, Chahynez Boubidi, Nadia Boukhenfouf, Mohamed Amine Ifri, Noureddine Khelafi, Houda Boudiaf, Tahar Khelifi Touhami, Fethi Meçabih, Malika Boucelma, Amara Zelaci, Ourida Gacem, Mohamed Samir Ladj, Azzedine Mekki, Nadia Bensaadi, Malika Benhalima, Zoulikha Zeroual, Belkacem Bioud, Mustapha Benameur, Rachid Bouhdjila, Zahir Bouzerar, Ouardia Ibsaine, Hachemi Maouche, Leila Kedji, Leila Smati, Rachida Boukari, Claude Lambert, Sergio D. Rosenzweig, Luigi D. Notarangelo, Kamel Djenouhat

**Affiliations:** ^1^ Department of Medical Biology, Rouiba Hospital, University of Algiers 1, Algiers, Algeria; ^2^ Department of Pediatrics, Mustapha University Hospital, University of Algiers 1, Algiers, Algeria; ^3^ Pediatric Gastroenterology, Centre Algérois de Pédiatrie, Algiers, Algeria; ^4^ Department of Pediatrics, Central Hospital of the Army, Algiers, Algeria; ^5^ Department of Pediatrics, Blida University Hospital, University of Blida, Blida, Algeria; ^6^ Department of Pediatrics, El-Harrach Hospital, University of Algiers 1, Algiers, Algeria; ^7^ Immunology Service, Department of Laboratory Medicine, Clinical Center, National Institutes of Health, Bethesda, MD, United States; ^8^ Department of Pediatrics, Ain Taya Hospital, University of Algiers 1, Algiers, Algeria; ^9^ Department of Pediatrics, Bab El-Oued University Hospital, University of Algiers 1, Algiers, Algeria; ^10^ Department of Pediatrics, Bologhine Hospital, University of Algiers 1, Algiers, Algeria; ^11^ Department of Pneumology, Rouiba Hospital, University of Algiers 1, Algiers, Algeria; ^12^ Department of Internal Medicine, Rouiba Hospital, University of Algiers 1, Algiers, Algeria; ^13^ Department of Pediatrics, Mansourah Hospital, University of Constantine, Constantine, Algeria; ^14^ Department of Pediatrics, Constantine University Hospital, University of Constantine, Constantine, Algeria; ^15^ Department of Pediatrics, Setif University Hospital, University of Setif, Setif, Algeria; ^16^ Department of Pediatrics, Meftah Hospital, Blida, Algeria; ^17^ Department of Pediatrics A, Hussein Dey University Hospital, University of Algiers 1, Algiers, Algeria; ^18^ Department of Pediatrics, Rouiba Hospital, Algiers, Algeria; ^19^ Department of Pediatrics, Thenia Hospital, Boumerdes, Algeria; ^20^ Department of Pediatric Oncology, Mustapha University Hospital, University of Algiers 1, Algiers, Algeria; ^21^ Private Practitioner, Constantine, Algeria; ^22^ Department of Immunology, Institut Pasteur d’Algérie, University of Algiers 1, Algiers, Algeria; ^23^ Department of Internal Medicine, Kouba Hospital, University of Algiers 1, Algiers, Algeria; ^24^ Department of Pediatrics, El Oued Hospital, El Oued, Algeria; ^25^ Department of Pediatrics, Birtraria Hospital El Biar, University of Algiers 1, Algiers, Algeria; ^26^ Department of Pediatrics B, Hussein Dey University Hospital, University of Algiers 1, Algiers, Algeria; ^27^ Department of Pediatrics, Tizi Ouzou University Hospital, University of Tizi Ouzou, Tizi Ouzou, Algeria; ^28^ Algiers Faculty of Pharmacy, University of Algiers 1, Algiers, Algeria; ^29^ Cytometry Unit, Immunology Laboratory, Saint-Etienne University Hospital, Saint-Étienne, Lyon, France; ^30^ National Institute of Allergy and Infectious Diseases, National Institutes of Health, Bethesda, MD, United States

**Keywords:** flow cytometry, lymphocyte phenotyping, protein expression assays, functional assays, flow cytometry-based diagnostic approach, complement deficiencies

## Abstract

**Purpose:**

In this study, we retrospectively reviewed the use of flow cytometry (FCM) in the diagnosis of inborn errors of immunity (IEIs) at a single center in Algeria. Sharing insights into our practical experience, we present FCM based diagnostic approaches adapted to different clinical scenarios.

**Methods:**

Between May 2017 and February 2024, pediatric and adult patients presenting with clinical features suggestive of immunodeficiency were subjected to FCM evaluation, including lymphocyte subset analysis, detection of specific surface or intracellular proteins, and functional analysis of immune cells.

**Results:**

Over a nearly seven-year period, our laboratory diagnosed a total of 670 patients (372 (55.5%) males and 298 (44.5%) females), distributed into 70 different IEIs belonging to 9 different categories of the International Union of Immunological Societies classification. FCM was used to diagnose and categorize IEI in 514 patients (76.7%). It provided direct diagnostic insights for IEIs such as severe combined immunodeficiency, Omenn syndrome, MHC class II deficiency, familial hemophagocytic lymphohistiocytosis, and CD55 deficiency. For certain IEIs, including hyper-IgE syndrome, STAT1-gain of function, autoimmune lymphoproliferative syndrome, and activated PI3K delta syndrome, FCM offered suggestive evidence, necessitating subsequent genetic testing for confirmation. Protein expression and functional assays played a crucial role in establishing definitive diagnoses for various disorders. To setup such diagnostic assays at high and reproducible quality, high level of expertise is required; in house reference values need to be determined and the parallel testing of healthy controls is highly recommended.

**Conclusion:**

Flow cytometry has emerged as a highly valuable and cost-effective tool for diagnosing and studying most IEIs, particularly in low-income countries where access to genetic testing can be limited. FCM analysis could provide direct diagnostic insights for most common IEIs, offer clues to the underlying genetic defects, and/or aid in narrowing the list of putative genes to be analyzed.

## 1 Introduction

Human inborn errors of immunity (IEIs) are a heterogenous group of monogenetic disorders characterized by an absent or aberrant function in one or more components of the immune system, which predisposes affected individuals to increased frequency and severity of infection, autoimmunity, allergy, and malignancy (1, 2). IEIs were traditionally viewed as rare disorders. However, recent reports suggest that IEIs are more common than previously believed, with a collective prevalence ranging from 1/5000 to 1/1000 (3). Furthermore, the wider usage of next-generation sequencing (NGS) platforms had greatly contributed to the discovery of new IEIs and broadened the spectrum of clinical phenotypes associated with IEI (4, 5).

The latest report from the International Union of Immunological Societies (IUIS) Expert Committee described 485 different disorders, categorized into ten groups (1). Most disorders in the IUIS classification have an autosomal recessive (AR) mode of inheritance, suggesting IEIs should be even more prevalent in consanguineous populations from North African countries like Algeria. However, the reported prevalence of ~1/50,000 in the Algerian registry remains low (6, 7). This means there is a significant underestimation of the burden of IEIs in Algeria. Several factors are likely culprits: insufficient awareness, a high frequency of severe forms–especially combined immunodeficiencies–linked to early mortality, and limitations in the availability of necessary diagnostic tools (6, 8).

The basic clinical and laboratory evaluation of a patient with a suspected IEI should encompass a detailed clinical history, a complete blood count (CBC), measurements of serum immunoglobulin (Ig) levels, and complement protein assays (9). Due to the heterogeneity of the clinical presentations of patients, diagnosing IEIs based solely on clinical and conventional laboratory findings can often be challenging. While more recently available genetic investigations serve as definitive tool for diagnosing IEIs, genetic tests are time-consuming, labor-intensive, and costly. Additionally, targeted single-gene tests may overlook causative mutations. Exome sequencing has recently garnered attention as a gold standard for diagnosing IEIs. However, exome sequencing still identifies causative genetic defects in only 40% to 58% of patients (10, 11). Moreover, establishing such a facility in the context of a developing country is highly challenging. Therefore, flow cytometry (FCM) emerges as a valuable and cost-effective tool bridging conventional laboratory testing and genetic testing. It provides a rapid and accurate results based on single-cell analysis (12).

Several authors have described the role of FCM in the diagnosis of IEIs; however, few have presented experiences from specific centers, and none from the Maghreb region (13–15). In this study, we retrospectively reviewed the use of FCM in the diagnosis of IEIs at a single center in Algeria, emphasizing the experiences of a diagnostic approach adapted to various clinical scenarios.

## 2 Materials and methods

The study was conducted at the department of medical biology in Rouiba hospital and received ethical approval from the local committee in accordance with the Declaration of Helsinki. The study was designed as a retrospective review of FCM’s use in diagnosing IEIs over a nearly seven-year period, from May 2017 to February 2024. All patients met the updated criteria of the European Society for Immunodeficiency (ESID) (www.esid.org) and were categorized according to the updated classification of the IUIS expert committee (1).

### 2.1 Patients’ enrollment and data collection

Peripheral blood samples were received from both pediatric and adult patients presenting with clinical features suggestive of immunodeficiency, such as repeated, unusual, or severe infections, as well as early-onset immune dysregulation conditions, including cytopenia, inflammatory bowel disease, and endocrinopathy. These samples were referred to our center from various pediatric, pneumology, internal medicine and infectious diseases departments affiliated with various public healthcare facilities spread across the whole country. Data were systematically collected for each patient using a standardized data form and entered into a computerized database. Requested information included patient’s demographics, family history, clinical history (including the age of onset and the main infectious and/or immune dysregulation manifestations; thanks to the close collaboration with clinicians), and laboratory and imaging findings.

### 2.2 Initial laboratory evaluation

Before delving into the exploration of primary immunodeficiency, we meticulously excluded the presence of any secondary immunodeficiencies, with a particular focus on HIV infection, as well as those induced by the use of drugs or renal and gastrointestinal losses. In addition to routine laboratory parameters, including complete differential blood counts, peripheral blood smear, serum immunoglobulin measurements, and complement protein assays, the initial assessment of IEI also included a basic immunophenotyping of circulating lymphocyte subsets, referred to as T-B-NK enumeration. This assay utilized six different markers to analyze various lymphocyte subsets, including CD3 as a pan-T marker, CD4 for T-helper cells, CD8 for T-cytotoxic cells, CD19 for B cells, and CD56/CD16 for NK cells. Lymphocytes were gated using CD45 *vs.* side scatter (SSc), and the percentages of different subpopulations were determined. FCM analysis were performed in a six-colors FACSCanto™ cytometer (BD Biosciences, US.) from 2017 to 2021 and in an eight color FACSLyric™ cytometer (BD Biosciences, US.) from 2022 to date.

### 2.3 Advanced flow cytometry evaluation

More advanced FCM analysis was performed when needed, based on clinical presentation (e.g., age of onset, location, types of infectious and autoimmune/inflammatory diseases etc.) and conventional laboratory findings. These specialized FCM tests include (**Table 1**):

**Table 1:** Lymphocyte phenotyping, disease specific protein analysis and functional assays for IEI diagnosis.

Test	Cell populations	Indications
Immunophenotyping of circulating lymphocytes		
Basic T-B-NK phenotyping	CD4+ T cells, CD8+ T cells, B cells, NK cells	Basic screening for IEI, CID, SCID, PAD
Extended T-cell phenotyping		
T1 panel	naïve (CD45RA^+^CCR7^+^), memory (CD45RO^+^) and CD8+ TEMRA (CD45RA^+^CCR7^−^)	SCID, OS, leaky SCID, CID, CVID
T2 panel	RTE (CD4^+^CD45RA^+^CD31^+^)	SCID, OS, leaky SCID, DiGeorge syndrome
T3 panel	Th1 (CXCR3^+^CCR6^−^), Th2 (CXCR3^−^CCR6^−^), Th17 (CXCR3^−^CCR6^+^), Tfh (CXCR5^+^)	HIES, CMC, CID
T4 panel	TCRαβ DNT (CD3^+^TCRαβ^+^CD4^−^CD8^−^)	ALPS, Patients with >7% of CD3+CD4-CD8- T cells
T5 panel	Treg (FoxP3^+^CD25^+^)	IPEX syndrome
Extended B-cell phenotyping		
B panel	switched memory (CD27^+^sIgD^−^), non-switched memory (CD27^+^sIgD^+^), transitional B cells (CD38^hi^CD24^hi^), plasmablasts (CD38^hi^CD24^−^) and CD21^lo^ B cells (CD38^low^CD21^low^)	CVID, HIMS, PAD, CID, LRBA deficiency, RIPK1 deficiency
Analysis of the expression of specific surface or intracellular proteins		
CD132	B cells	T^−^B^+^NK^-^ SCID
CD127	T cells	T^−^B^+^NK^+^ SCID
HLA-DR	B cells	CID with low CD4 (MHC-II deficiency)
HLA-ABC	lymphocytes	CID with low CD8 (MHC-I deficiency)
ZAP-70	T cells	CID with low CD8
CD40L	PMA/Ionomycin stimulated CD4+ T cells	X-linked HIMS
CD40	B cells	HIMS
WASp	T cells	WAS
Btk	monocytes	XLA
Perforin	NK cells	FLH 2
CD18/CD11a	neutrophils	LAD-1
CD15	neutrophils	LAD-2
IFN-γR1 (CD119)	monocytes	MSMD
IL-12Rβ1 (CD212)	PHA stimulated T cells	MSMD
MCP/CD46	lymphocytes	aHUS
DAF/CD55	neutrophils	PLE (CHAPLE disease)
Functional assays		
Oxidative burst assay (DHR 123 assay)	neutrophils	CGD, EO-IBD
IL-17/IFN-γ production	PMA/Ionomycin stimulated CD4+ T cells	HIES, CMC
STAT3/STAT1 phosphorylation assays	IL6/IFN-γ stimulated T cells/monocytes	HIES, MSMD, CMC
NK-cell degranulation assay	PMA/Ionomycin/Brefeldin stimulated CD3^−^CD56^+^ NK cells	FLH 3, 4 and 5, CHS, GS2

aHUS, atypical hemolytic uremic syndrome; ALPS, autoimmune lymphoproliferative syndrome; Btk, Bruton’s tyrosine kinase; CGD, chronic granulomatous disease; CID, combined immunodeficiency; CHAPLE, complement hyperactivation angiopathic thrombosis and protein-losing enteropathy; CHS, Chediak-Higashi syndrome; CMC, chronic mucocutaneous candidiasis; CVID, common variable immunodeficiency; DAF, decay accelerating factor; DHR, Dihydrorhodamine; EO-IBD, early onset inflammatory bowel disease; FHL, familial hemophagocytic lymphohistiocytosis; GS2, Griscelli syndrome type 2; HIMS, hyper-IgM syndrome; HIES, hyper-IgE syndrome; HLA, human leukocyte antigen; IEI, inborn errors of immunity; IPEX, immune dysregulation-polyendocrinopathy-enteropathy-x-linked; LRBA, LPS-responsive beige-like anchor protein; LAD, leukocyte adhesion deficiency; MCP, membrane cofactor protein; MHC, major histocompatibility complex; MSMD, mendelian susceptibility to mycobacterial disease, OS, Omenn syndrome, PAD, predominantly antibody deficiency; PHA, phytohemagglutinin; PLE, protein losing enteropathy; PMA, phorbol-12-myristate-13-acetate; RIPK1, receptor-interacting serine/threonine-protein kinase 1; RTE, recent thymic emigrants; SCID, severe combined immunodeficiency; sIgD, surface IgD; TEMRA, terminally differentiated effector memory CD45RA+; Treg, regulatory T cells; WAS, Wiskott Aldrich syndrome; ZAP-70, Zeta-associated protein 70.

i. Extended phenotyping of circulating T- and B-cell subsets;
ii. Analysis of the expression of specific surface or intracellular proteins;
iii. Functional assays, including cytokine production, STAT1 and STAT3 phosphorylation, and NK-cell degranulation assays. Detailed methods can be found in this article’s Online Repository at www.frontiersin.org.

#### 2.3.1 Extended phenotyping of T- and B-cell subsets

##### 2.3.1.1 Extended T-cell phenotyping

A detailed analysis of naive and memory T-cell subpopulations was conducted in all patients with T lymphopenia and/or an initial clinical and laboratory evaluation suggestive of Omenn syndrome (OS), atypical severe combined immunodeficiency (SCID) or CID. A six-color panel (T1 panel) was used for this purpose, incorporating CD3, CD4, CD8, CD45RA, CD45RO, and CCR7 (CD197) markers, enabling the identification of naïve (CD45RA+CCR7+), memory (CD45RO+), and terminally differentiated effector memory CD45RA+ (TEMRA) among CD4+ or CD8+ T cells (**Table 1**, **Supplementary Table S1**). In some cases, CD31 (PECAM-1) was used as a marker to aid in the identification of CD4+ recent thymic emigrants (RTE) expressing CD45RA and CD31 markers (T2 panel) (16, 17).

In addition, other panels have been designed to identify further specific T-cell subpopulations (**Supplementary Table S1**). In patients with suspected hyper-IgE syndrome (HIES), Th17 cells were evaluated by a surface staining of memory CD4+ T cells (gated on CD45RO *vs.* CD4 dot plot), measuring membrane expression of CXCR3 (CD183) and CCR6 (CD196) chemokine receptors (T3 panel). Th17 cells exhibit the following profile: CD45RO^+^CXCR3^-^CCR6^+^ (18). TCRαβ double-negative T cells (TCRαβ DNT) were estimated in patients with suspected autoimmune lymphoproliferative syndrome (ALPS) or elevated CD3^+^CD4^-^CD8^-^ T cells, using the flowing panel: TCRαβ, TCRγδ, CD3, CD4, and CD8 (T4 panel).

Regulatory T cells (Treg) were identified either by the analysis of intracellular FoxP3 expression of or by surface staining of CD4, CD45RA, CD25, CD127 markers in suspected cases of Immune Dysregulation, Polyendocrinopathy, Enteropathy X-Linked (IPEX) Syndrome (T5 and T5bis panel).

##### 2.3.1.2 Extended B-cell phenotyping

Extended phenotyping of circulating B cells was performed in case of suspected predominantly antibody deficiency (PAD) with normal to moderately decreased B cells (B cells >2%). A multicolor panel, incorporating CD19, CD27, sIgD, CD24, CD38, and CD21 markers was used to analyze different B-cell subsets, including naïve (CD27^-^sIgD^+^), switched memory (CD27^+^sIgD^-^), non-switched memory (CD27^+^sIgD^+^), transitional (CD38^hi^CD24^hi^), plasmablasts (CD38^hi^CD24^-^) and CD21-low (CD21^lo^) B cells (CD38^low^CD21^low^) (**Supplementary Table S1**).

#### 2.3.2 Analysis of the expression of specific surface or intracellular proteins

##### 2.3.2.1 Cell surface proteins

Flow cytometry was used to evaluate specific cell surface proteins, targeted based on the suspected diagnosis (**Table 1**). Both percentage and mean/median fluorescence intensity (MFI) were determined and compared to controls. Common γ chain (CD132) and IL-7RA (CD127) expression were evaluated in patients with suspected X-linked (XL)-SCID (T-B+NK-SCID) and T-B+NK+SCID, respectively. Patients with suspected CID were systematically screened for major histocompatibility complex class II (MHC-II) deficiency by the assessment of human leukocyte antigen-DR (HLA-DR) on B cells and monocytes. MHC class I expression on lymphocytes was performed in patients with isolated CD8 lymphopenia. For patients with suspected hyper-IgM syndrome (HIMS), we studied CD40 expression on B cells (AR-HIMS) and CD40L (CD154) expression on CD4+ T cells after stimulation with phorbol myristate acetate (PMA) and ionomycin (X-linked HIMS). FCM-based diagnosis of LAD-1 and LAD-2 included the assessment of CD18/CD11a (LAD-1) and CD15 (LAD-2) expression on gated neutrophils. IFN-γR1 (CD119) and IL-12Rβ1 (CD212) were analyzed in patients with a picture suggestive of mendelian susceptibility to mycobacterial disease (MSMD). Patients with atypical hemolytic uremic syndrome (aHUS) and early-onset protein-losing enteropathy (PLE) were screened for CD46/MCP (membrane cofactor protein) deficiency and CD55/DAF (decay accelerating factor) deficiency, respectively (**Table 1**).

##### 2.3.2.2 Intracytoplasmic proteins

Selected intracytoplasmic proteins were analyzed on permeabilized lymphocytes or monocytes according to the suspected diagnosis (**Table 1**). Isotypic controls (IC) were used to calculate a staining index, defined as the ratio of the MFI of the targeted protein to that of the IC. The staining index was determined for both patients and controls. In patients with suspected Wiskott–Aldrich syndrome (WAS), intracytoplasmic staining for WAS protein (WASp) was performed using purified mouse anti-human WASp monoclonal antibody and secondary FITC-conjugated anti-Mouse IgG2a. WASp expression was evaluated on T cells. ZAP-70 staining on T cells was performed in patients with isolated CD8 lymphopenia. For male patients with suspected X-Linked Agammaglobulinemia (XLA), Bruton’s tyrosine kinase (Btk) protein expression analysis was carried out on permeabilized CD14+ monocytes. Patients with hemophagocytic lymphohistiocytosis (HLH) were screened for perforin deficiency (i.e., Familial hemophagocytic lymphohistiocytosis type 2 or FHL2) by analyzing the expression of perforin on permeabilized CD3^-^CD56^+^NK cells.

#### 2.3.3 Functional assays

##### 2.3.3.1 Dihydrorhodamine 123 assay

DHR 123 assay was used for the diagnosis of chronic granulomatous disease (CGD). Patient’s neutrophils were stimulated for 20 minutes with PMA in the presence of DHR 123. A stimulation index (SI) was calculated for gated neutrophils (SI: the ratio of the MFI of the stimulated to the unstimulated neutrophils). SI of 100 was considered the cutoff in our laboratory.

##### 2.3.3.2 Cytokines production assay

In patients with picture of HIES or chronic mucocutaneus candidiasis (CMC), IL-17A and IFN-γ production was measured following stimulation of whole blood or peripheral blood mononuclear cells (PBMC) with PMA/Ionomycin in the presence of protein transport inhibitor (Brefeldin).

##### 2.3.3.3 Signal transducers and activators of transcription phosphorylation assays

STAT3 phosphorylation was evaluated on CD4+ T cells after stimulation of PBMC with IL-6 followed by fixation and permeabilization of the cells and incubation with anti-pSTAT3. A SI was calculated for gated CD4+ T cells by dividing the MFI of stimulated cells by the MFI of unstimulated cells. STAT1 phosphorylation after IFN-γ stimulation was evaluated in patients with suspected MSMD or CMC due to gain of function (GOF) mutation in STAT1.

##### 2.3.3.4 NK-cell degranulation assay

The degranulation assay of resting NK cells was conducted in patients with HLH and normal perforin expression, along with those suspected of Chediak–Higashi syndrome (CHS) or Griscelli syndrome type 2 (GS2). After two hours stimulation of whole blood or PBMC with PMA and ionomycin, CD3^-^CD56^+^NK cells were gated and assessed for surface expression of CD107a. ΔCD107a, which is the difference of surface CD107a expression between stimulated and non-stimulated NK cells, was determined. Defective degranulation was arbitrarily defined as less than 5% degranulation, whereas abnormal degranulation was defined as being lower than 10%.

### 2.4 Genetic testing

Genetic testing is not routinely available in our laboratory. When feasible, patients with MHC-II deficiency underwent screening for the 752delG26 mutation (deletion of 26bp) by analyzing the size of PCR products by electrophoresis in a 2% agarose gel. Sanger sequencing was performed on specific genes including, *RAG1*, *RAG2*, *ADA*, *IL2RG*, *CD40, DOCK8*, *WAS, BTK, LYST*, *ITGB2, C3, and CD46*. Next generation sequencing (NGS) through targeted gene sequencing panels or whole exome/genome sequencing was performed in patients with undefined clinical and/or immunological picture.

## 3 Results

### 3.1 Baseline characteristics

**Figure 1:**
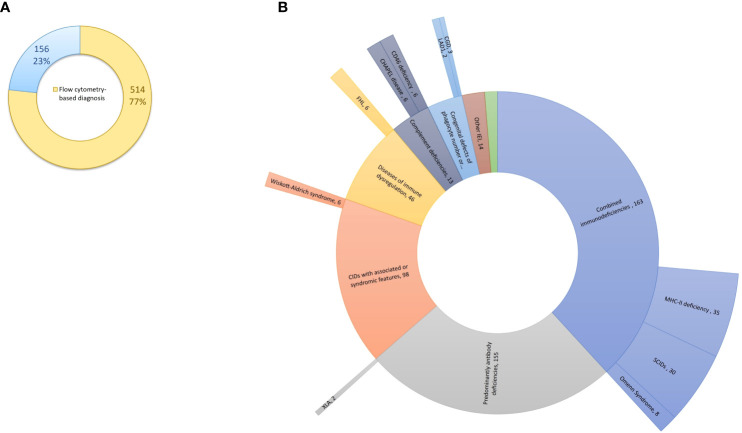
Flow cytometry-based diagnosis of IEI at our center. **(A)** Flow cytometry has proven instrumental in diagnosing and categorizing IEI in 514 patients (77%). **(B)** Among the 514 patients (internal circle), flow cytometry analysis led to the definitive diagnosis in 104 cases (external circle). IEI, inborn errors of immunity.

Between May 2017 and February 2024, a total of 4,277 patients underwent screening for IEI at our center. Among them, 670 patients, comprising 372 (55.5%) males and 298 (44.5%) females, were diagnosed with IEI. The median age of IEI patients at time of diagnosis was 6 years (0.1 – 81 years), and the median age at the onset of symptoms was 1 year (0 – 66 years). Four hundred eighty-seven patients (72.7%) were children, while 183 (27.3%) were adults. Nearly half of patients (47.5%) were born from consanguineous parents. FCM helped to categorize and reach the diagnosis in 514 patients (76.7%), while 156 (23.3%) were categorized based on other laboratory parameters (e.g., measurement of serum complement C4 and C1-inhibitor in hereditary angioedema), clinical manifestations and/or genetic testing (**Supplementary Table S2**). Of the 514 patients, flow cytometry analysis yielded a definitive diagnosis in 104 cases. For the remaining patients, flow cytometry proved instrumental in categorizing IEI into functional groups and provided valuable insights into the underlying genetic defect, albeit requiring subsequent genetic testing for confirmation (**Figure 1**). Genetic analysis was performed in 118 patients, revealing the genetic defect in 93 (78.8%) patients. The distribution of patients according to the classification from the IUIS expert committee is shown in **Table 2** and **Supplementary Figure S1**. Combined immunodeficiencies (24.3%) were the most common, followed by predominantly antibody deficiencies (23.1%) and complement deficiencies (22.8%). Hereby we present the results of the patients screened and/or diagnosed based on FCM results.

**Table 2:** Distribution and laboratory findings of IEIs in our series.

Category	Disease	N (%)	Age at diagnosis (months) median (range)	Associated flow cytometry findings	
				*Findings*	*%*
**Combined immunodeficiencies**		163 (24.3%)	17 (1 – 552)		
	SCIDs	30 (4.5%)	5 (2 – 17)	T cells < 300/μl	96.7%
	Omenn Syndrome	8 (1.2%)	4.5 (1 – 10)	Absent naïve T cells Absent RTE Absent B cells	100% 100% 100%
	Leaky SCIDs	9 (1.3%)	19 (5 – 240)	Decreased naïve T cells Very low RTE Reduced B cells	100% 100% 88.9%
	MHC-II deficiency	35 (5.2%)	20 (1 – 132)	Absent HLA-DR expression on B cells CD4 lymphopenia Inverted CD4/CD8 ratio	100% 88.6% 74.3%
	Other CID	81 (49.7%)	36 (2 – 552)	CD4 lymphopenia Inverted CD4/CD8 ratio Decreased naïve CD8 T cells Decreased naïve CD4 T cells	74.1% 60.5% 95.8%^*^ 81.9%^*^
**CIDs with associated or syndromic features**		101 (15.1%)	48 (1 – 204)		
	DiGeorge syndrome	7 (1.0%)	3 (2 – 11)	CD3 lymphopenia	100%
	Ataxia Telangiectasia	11 (1.6%)	90 (30 – 204)	CD3 lymphopenia Decreased naïve T cells	100% 100%
	Wiskott-Aldrich syndrome	16 (2.4%)	8 (1 – 48)	CD4 lymphopenia Inverted CD4/CD8 ratio Absent/reduced WASp expression	62.5% 62.5% 66.7%^**^
	Hyper-IgE syndrome	57 (8.5%)	60 (8 – 192)	Defective IL-17 production Low CXCR3^−^CCR6^+^ Th17 cells Absent/reduced STAT3 phosphorylation	81.8%^#^ 45.5%^#^ 9.1%^#^
	ARPC1B deficiency	2 (0.3%)	96 (72 – 120)	Hyper-IgE CD3 lymphopenia Decreased naïve T cells Expanded TCRαβ DNT	100% 100% 100% 50%
**Predominantly antibody deficiencies**		155 (23.1%)	96 (5 – 804)		
	Agammaglobulinemia	17 (2.5%)	36 (5 – 312)	Severe decrease of sIg Absent/profoundly decreased B cells Absent Btk expression (in male patients)	100% 100% 100%**^¥^**
	CVID	70 (10.4%)	252 (18 – 804)	Reduced switched memory B cells Expanded transitional B cells Expanded CD21^lo^ B cells	80.9%**^§^** 23.8%**^§^** 28.6%**^§^**
	Activated p110δ syndrome	2 (0.3%)	21 (18 – 24)	Reduced switched memory B cells Expanded transitional B cells Decreased naïve T cells	100% 100% 100%
	SIAD	15 (2.2%)	72 (30 – 624)		
	Unclassified antibody deficiency	53 (7.9%)	48 (5 – 708)		
**Diseases of immune dysregulation**		49 (7.3%)	48 (1 – 336)		
	Chediak-Higashi Syndrome	9 (1.3%)	30 (1 – 84)	Defective NK-cell degranulation assay	100%**^£^**
	Griscelli Syndrome	1 (0.1%)	72		
	FHL	7 (1.0%)	14 (1 – 54)	Defective/abnormal NK-cell degranulation	71.4%
	ALPS	5 (0.7%)	42 (10 – 132)	Increased TCRαβ DNT	100%
	RIPK1 deficiency	4 (0.6%)	78 (48 – 144)	Hypo-IgA and hypo-IgM Reduced switched memory B cells Elevated transitional B cells CD4 lymphopenia	100% 100% 100% 100%
	LRBA deficiency	3 (0.4%)	216 (156 – 336)	Hypogammaglobulinemia Reduced switched memory B cells Expanded CD21^lo^ B cells	100% 100% 100%
	TPP2 deficiency	2 (0.3%)	106 (32 – 180)	Hypogammaglobulinemia Reduced naïve T cells	100% 100%
	IPEX syndrome	1 (0.1%)	17	Markedly decreased FoxP3^+^CD25^+^ regulatory T cells	100%
**Congenital defects of phagocyte number or function**		20 (3.0%)	23 (1 – 372)		
	Congenital neutropenia	8 (1.2%)	22.5 (3 – 36)		
	Cyclic neutropenia	2 (0.3%)	26.5 (23 – 30)		
	CGD	7 (1.0%)	36 (5 – 372)	Absent/markedly reduced NADPH oxidase activity	100%
	LAD-1	2 (0.3%)	9.5 (1 – 18)	Absent CD18/CD11a expression	100%
**Defects in intrinsic and innate immunity**		12 (1.8%)	66 (18 – 252)		
	MSMD	4 (0.6%)	78 (30 – 252)		
	CMC	5 (0.7%)	36 (24 – 72)	IL-17A production defect Hyperphosphorylation of STAT1 after IFN-γ stimulation	100% 100%
**Autoinflammatory disorders**		2 (0.3%)	84 (72 – 96)		
	Blau syndrome	1 (0.1%)	72		
	A20 deficiency	1 (0.1%)	96		
**Complement deficiencies**		153 (22.8%)	288 (12 – 972)		
	C1 inhibitor deficiency	115 (17.2%)	336 (12 – 972)		
	Factor I deficiency	5 (0.7%)	426 (252 – 552)		
	Factor H deficiency	3 (0.4%)	348 (12 – 468)		
	CD46 deficiency	7 (1%)	240 (144 – 456)	Absent CD46 expression on lymphocytes Reduced CD46 expression on lymphocytes	85.7% 14.3%
	C3 deficiency	2 (0.3%)	96 (72 – 120)		
	C7 deficiency	1 (0.1%)	264		
	CHAPLE disease	6 (0.9%)	72 (24 – 168)	Absent CD55 expression on neutrophils	100%
**Phenocopies of IEI**	Good syndrome	3 (0.4%)	780 (636 – 828)	Hypogammaglobulinemia Profoundly decreased B cells Inverted CD4/CD8 ratio	100% 100% 100%
**Unclassified immunodeficiencies**		12 (1.8%)	36 (7 – 516)		

^*^: naïve and memory T cells were analyzed in 72 patients.

^**^: WASp expression was assessed in 9 patients.

^#^: Th17, Il-17 production and STAT3 phosphorylation were assessed in 22 patients.

^¥^: Btk expression was assessed in 2 male patients.

**^§^**: Phenotyping of circulating B-cell subpopulations was performed in 63 patients.

**^£^**: NK-cell degranulation assay was performed in two patients.

ALPS, autoimmune lymphoproliferative syndrome; APDS, activated PI3K delta syndrome; ARPC1B, actin related protein 2/3 complex subunit 1B; Btk, Bruton’s tyrosine kinase; CGD, chronic granulomatous disease; CID, combined immunodeficiency; CHAPLE, complement hyperactivation angiopathic thrombosis and protein-losing enteropathy; CMC, chronic mucocutaneous candidiasis; CVID, common variable immunodeficiency; FHL, familial hemophagocytic lymphohistiocytosis; HLA, human leukocyte antigen; IPEX, immune dysregulation-polyendocrinopathy-enteropathy-x-linked; LRBA, LPS-responsive beige-like anchor protein; MHC, major histocompatibility complex; MSMD, mendelian susceptibility to mycobacterial disease, OS, Omenn syndrome; RIPK1, receptor-interacting serine/threonine-protein kinase 1; RTE, recent thymic emigrants; SCID, severe combined immunodeficiency; SIAD, selective IgA deficiency; sIg, serum immunoglobulins; TPP2, tripeptidyl peptidase 2; STAT, signal transducer and activator of transcription; WASp, Wiskott Aldrich syndrome protein.

### 3.2 Combined immunodeficiencies

Combined immunodeficiencies were the most common with 163 cases, including 30 (18.4%) SCID, 8 (4.9%) Omenn syndrome, 9 (5.5%) leaky SCID, and 35 (21.5%) MHC class II deficiency (**Table 2**). The diagnosis was made based on FCM analysis in all patients; genetic analysis was carried out in 54 (33.1%) patients, leading to the identification of the genetic defect in 40 (24.5%) patients (**Table 3**).

**Table 3:** IEI causing genes identified in our series.

Category	Disease	Inheritance	Gene	Number of patients
**Combined immunodeficiencies**	T^−^B^+^ SCID	XL	*IL2RG*	1
	T^−^B^−^ SCID	AR	*RAG1*	2
		AR	*RAG2*	1
		AR	*ADA*	1
	Omenn Syndrome	AR	*RAG1*	4
		AR	*RAG2*	1
	T^low^B^–/low^ leaky SCID	AR	*RAG1*	2
		AR	*RAG2*	2
		AR	*DCLRE1C*	1
		AR	*LIG4*	1
		AR	*ADA*	1
	T^low^B^+^ leaky SCID	AR	*CORO1A*	2
	MHC-II deficiency	AR	*RFXANK*	10
	MHC-I deficiency	AR	*TAP2*	1
	HIMS	AR	*CD40*	2
	CD3γ deficiency	AR	*CD3G*	2
	FCHO1 deficiency	AR	*FCHO1*	2
	DOCK8 deficiency	AR	*DOCK8*	1
	SASH3 deficiency	XL	*SASH3*	1
	IKBKB deficiency	AR	*IKBKB*	1
	HELIOS deficiency	AR	*IKZF2*	1
**CIDs with associated or syndromic features**	Wiskott-Aldrich syndrome	XL	*WAS*	2
	Arp2/3-mediated filament branching defect	AR	*ARPC1B*	2
	AD-HIES STAT3 deficiency (Job syndrome)	AD	*STAT3*	1
	Vici syndrome	AR	*EPG5*	1
**Predominantly antibody deficiencies**	XLA	XL	*BTK*	7
	Activated p110δ syndrome	AD	*PIK3CD GOF*	2
	TACI deficiency	AD	*TNFRSF13B*	1
**Diseases of immune dysregulation**	Chediak-Higashi Syndrome	AR	*LYST*	7
	Munc13–4 deficiency (FHL3)	AR	*UNC13D*	1
	LRBA deficiency	AR	*LRBA*	3
	RIPK1 deficiency	AR	*RIPK1*	4
	Tripeptidyl-Peptidase II Deficiency	AR	*TPP2*	2
	ALPS-FAS	AR	*TNFRSF6*	2
**Defects in intrinsic and innate immunity**	MSMD	AR	*IL12RB1*	2
		AR	*TYK2*	1
	CMC	AD	*STAT1 GOF*	2
**Autoinflammatory disorders**	Blau syndrome	AD	*NOD2*	1
	A20 deficiency	AD	*TNFAIP3*	1
**Complement deficiencies**	C3 deficiency	AR	*C3*	2
	CD46 deficiency	AR	*CD46*	3
	CHAPLE disease	AR	*CD55*	6

ALPS, autoimmune lymphoproliferative syndrome; ARPC1B, actin related protein 2/3 complex subunit 1B; CHAPLE, complement hyperactivation angiopathic thrombosis and protein-losing enteropathy; CMC, chronic mucocutaneous candidiasis; FCHO1, F-BAR domain only protein 1; FHL, familial hemophagocytic lymphohistiocytosis; HIMS, hyper-IgM syndrome; HIES, hyper-IgE syndrome; IKBKB, inhibitor of nuclear factor kappa-B kinase, subunit beta; LRBA, LPS-responsive beige-like anchor protein; MHC, major histocompatibility complex; MSMD, mendelian susceptibility to mycobacterial disease; RIPK1, receptor-interacting serine/threonine-protein kinase 1; SCID, severe combined immunodeficiency; TACI, transmembrane activator and CAML interactor; WAS, Wiskott Aldrich syndrome; XLA, X-linked agammaglobulinemia.

#### 3.2.1 Severe combined immunodeficiencies

Of the 30 patients with SCID, 14 (46.7%) were male and 16 (53.3%) were females. The median age at diagnosis was 5 months (range: 2 – 17) and the median age at symptoms onset was one month (range: 0.1 – 4). All but one patient had less than 300 T cells/μl and the mean T-cell count was 58/μl (range: 0 – 545). Twenty-one (70%) were diagnosed with T-B-NK+ SCID, 5 (16.7%) with T-B+NK+ SCID, 3 (10%) with T-B+NK- SCID, and one (3.3%) with T-B-NK- SCID. Common γ chain expression was evaluated in two male patients with T-B+NK-SCID and was defective in one of them. IL-7RA expression was assessed in one T-B+NK+ SCID patient and was normal. Sanger sequencing was performed based on FCM classification in 5 patients revealing homozygous *RAG1*/*RAG2* variants in 3 patients, and homozygous *ADA* mutation and hemizygous *IL2RG* variant in the remaining two patients (**Table 3**).

#### 3.2.2 Omenn syndrome and leaky SCIDs

Eight patients (4 males and 4 females) were diagnosed with OS. The median age at diagnosis was 4.5 months. All patients exhibited T^low/norm^B-NK+ profile with T-cell count ranging from 340/μl to 18360/μl (median=533/μl). Circulating B cells were absent in all patients, while NK-cell count ranged from 298 to 2123/μl (median= 715/μl). The T-cell subpopulation analysis was notable for the absence of naïve T cells and RTE in all patients (**Table 2**). Genetic analysis was performed in 5 patients revealing homozygous mutations of *RAG1* in four patients and a homozygous variant of *RAG2* in another one (**Table 3**).

**Figure 2:**
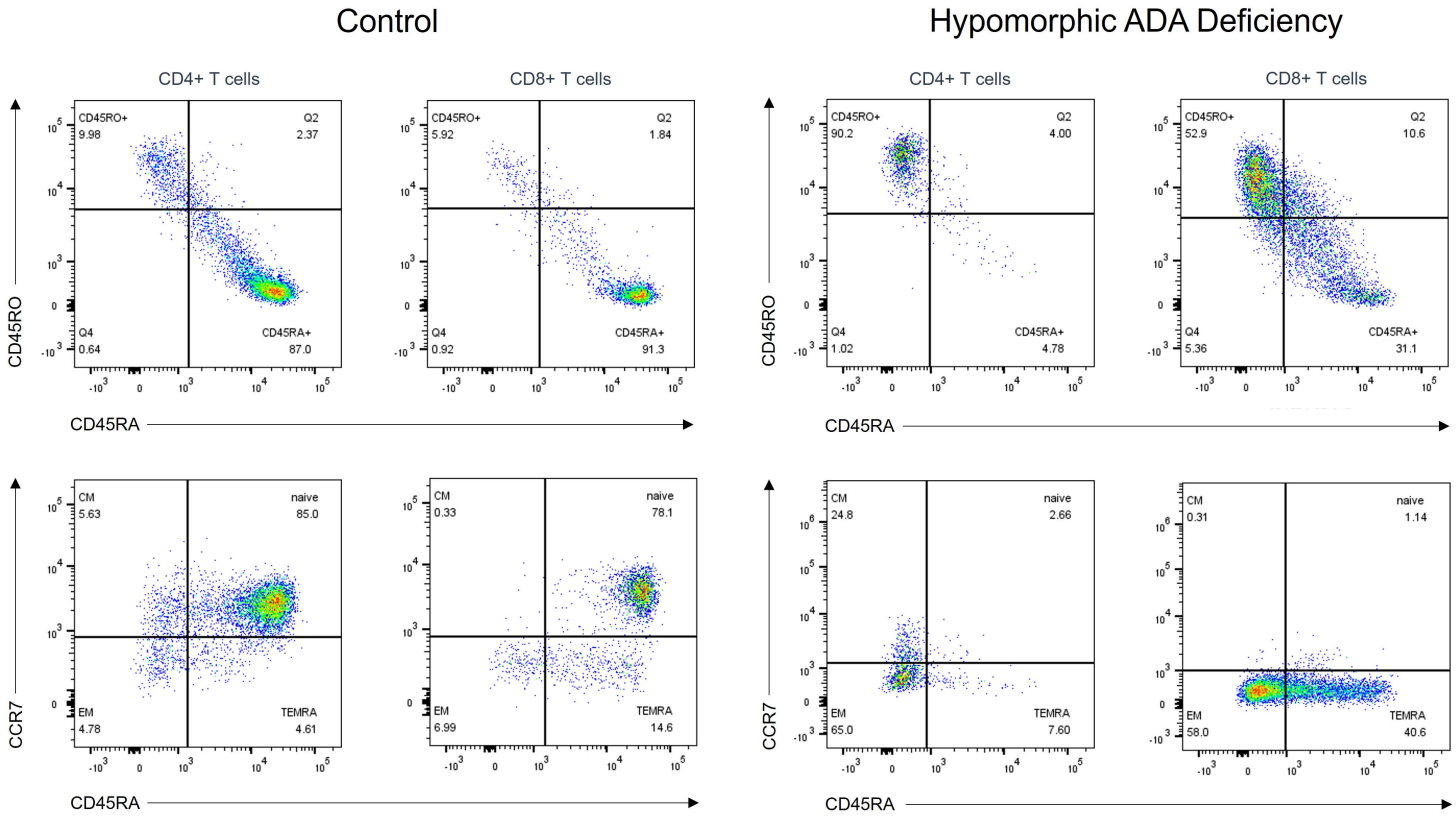
T-cell subpopulation analysis in a patient with hypomorphic ADA deficiency and an age-matched healthy control. T cells from the patient, carrying a homozygous ADA variant (c.965T>C= p.Phe322Ser), exhibit a memory phenotype (CD45RO+) with nearly absent native (CD45RA+CCR7+) subsets. ADA, adenosine deaminase.

Nine patients (4 males and 5 females) carried homozygous hypomorphic variants in *RAG1* (n=2), *RAG2* (n=2), *DCLRE1C* (n=1), *LIG4* (n=1), *ADA* (n=1), and *CORO1A* (n=2), and presented with leaky SCID (i.e., 7 patients with T^low^ B^-/low^ leaky SCID and 2 patients with T^low^ B^+^ leaky SCID). The median age at diagnosis was 19 months (range: 5 – 240) and the median age of symptoms onset was 6 months (range: 4 – 60). The median T-cell count was 614/μl (range: 471 – 2530) and the percentage of CD4+CD45RA+ and CD8+CD45RA+CCR7+ T cells ranged from 0.7% to 25.0% (median=6.5%) and 0.0% to 19.0% (median=6.3%), respectively. RTE were very low in all patients with a median of 1.7% (range 0% – 7.5%). Among patients with T^low^ B^-/low^ leaky SCID, B-cell count was decreased or markedly decreased in all patients, with a median of 24/μl (range: 4 – 261) (**Figure 2**).

#### 3.2.3 Bona fide CIDs

One hundred sixteen patients were diagnosed with CID based on FCM analysis. CD3 lymphopenia was seen in 61 (52.6%) patients, CD4 and CD8 lymphopenia were present in 91 (78.4%) and 43 (37.1%) patients respectively, while inverted CD4/CD8 ratio was noted in 75 (64.7%) patients. Extended analysis of T-cell subpopulations was performed in 90 (77.6%) patients, revealing expanded γδ T cells in 8 (8.9%) patients, and reduced naïve CD4 and CD8 T cells in 76 (84.4%) and 79 (87.8%) patients, respectively. Genetic testing was performed in 35 patients revealing 9 different disorders, i.e., RFXANK, CD40, DOCK8, CD3γ, FCHO1, SASH3, IKBKB, HELIOS, and TAP2 deficiencies, in 21 patients (**Table 3**). CD3γ deficiency was diagnosed in two siblings presenting with recurrent infections (recurrent pneumonia, multiple abscesses and candidiasis), lymphoproliferation and autoimmune hemolytic anemia. Interestingly, FCM analysis was notable for quantitative variations of circulating T and B cells (T-cell lymphopenia, markedly decreased naïve T cells, and expanded CD21^lo^ B cells), but also for low expression of the TCR-CD3 complex on the T-cell surface.

MHC-II deficiency was the most frequent CID in our series with 35 cases, representing 21.5% of the total number of CIDs. Typically, MHC-II deficiency is characterized by hypogammaglobulinemia, CD4 lymphopenia and absent HLA-II expression on B cells and monocytes. In our series, low CD4 T-cell count was seen in 31 (88.6%) patients, inverted CD4/CD8 ratio in 26 (74.3%) patients, and CD8 lymphopenia in 10 (28.6%) patients. Decreased IgG, IgA and/or IgM were noted in 19 (54.3%), 26 (74.3%), and 15 (42.9%) patients, respectively. HLA-DR expression at the surface of B cells was absent in 34 patients and markedly decreased in one patient.

### 3.3 CIDs associated with syndromic features

#### 3.3.1 Wiskott-Aldrich syndrome

Sixteen patients were diagnosed with WAS in our center. Thirteen patients (81.2%) showed decrease in CD4 T cells, CD8 T cells or both. CD4/CD8 ratio was low in 10 patients (62.5%) and high in 4 (25%). Intracytoplasmic staining for WASp was performed in 9 patients unveiling reduced or absent WASp expression in six patients.

#### 3.3.2 Hyper-IgE syndrome

**Figure 3:**
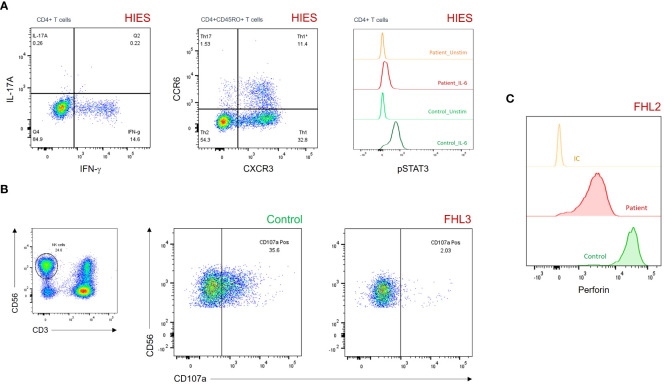
**(A)** Dot plots and histograms showing reduced IL-17A production (upper left panel), CD45RO^+^CCR6^+^CXCR3^–^ Th17 cells (upper middle panel), and pSTAT3 levels after IL-6 stimulation (upper right panel) in a patient with HIES. **(B)** Degranulation assay of resting NK cells showing impaired CD107a surface expression in a patient with FHL3. **(C)** Histograms showing partial perforin expression defect on gated CD3^–^CD56^+^ NK cells in a patient with FHL2. FHL, familial hemophagocytic lymphohistiocytosis; HIES, hyper-IgE syndrome; IC, isotypic control; pSTAT3, phosphorylated signal transducer and activator of transcription 3.

Fifty-seven patients had hyper-IgE (i.e., IgE > 10 times the norm for age) and pathologic susceptibility to infections with no evidence of T-cell or B-cell deficiency. They were diagnosed with HIES according to ESID criteria. Th17 cells, IL-17 production and STAT3 phosphorylation were assessed in 22 patients. Defective IL-17 production was found in 18 (81.8%) patients, low Th17 cells in 10 (45.5%) patients, while a reduced or absent STAT3 phosphorylation assay was observed in two (9.1%) patients (**Table 2**, **Figure 3**).

### 3.4 Predominantly antibody deficiencies

One hundred fifty-five patients were diagnosed with PAD. Among them, 17 (11.0%) had Agammaglobulinemia, 70 (45.2%) had common variable immunodeficiency (CVID), and 15 (9.7%) had selective IgA deficiency (**Table 2**).

#### 3.4.1 Agammaglobulinemia

Seventeen patients, including 13 males and 4 females, were diagnosed with Agammaglobulinemia. Sequencing of the *BTK* gene was performed in ten male patients. Seven of them (70%) had hemizygous pathogenic variants in *BTK*. Intracytoplasmic Btk staining was carried out in two patients, both of them displayed absent Btk expression.

#### 3.4.2 CVID

**Figure 4:**
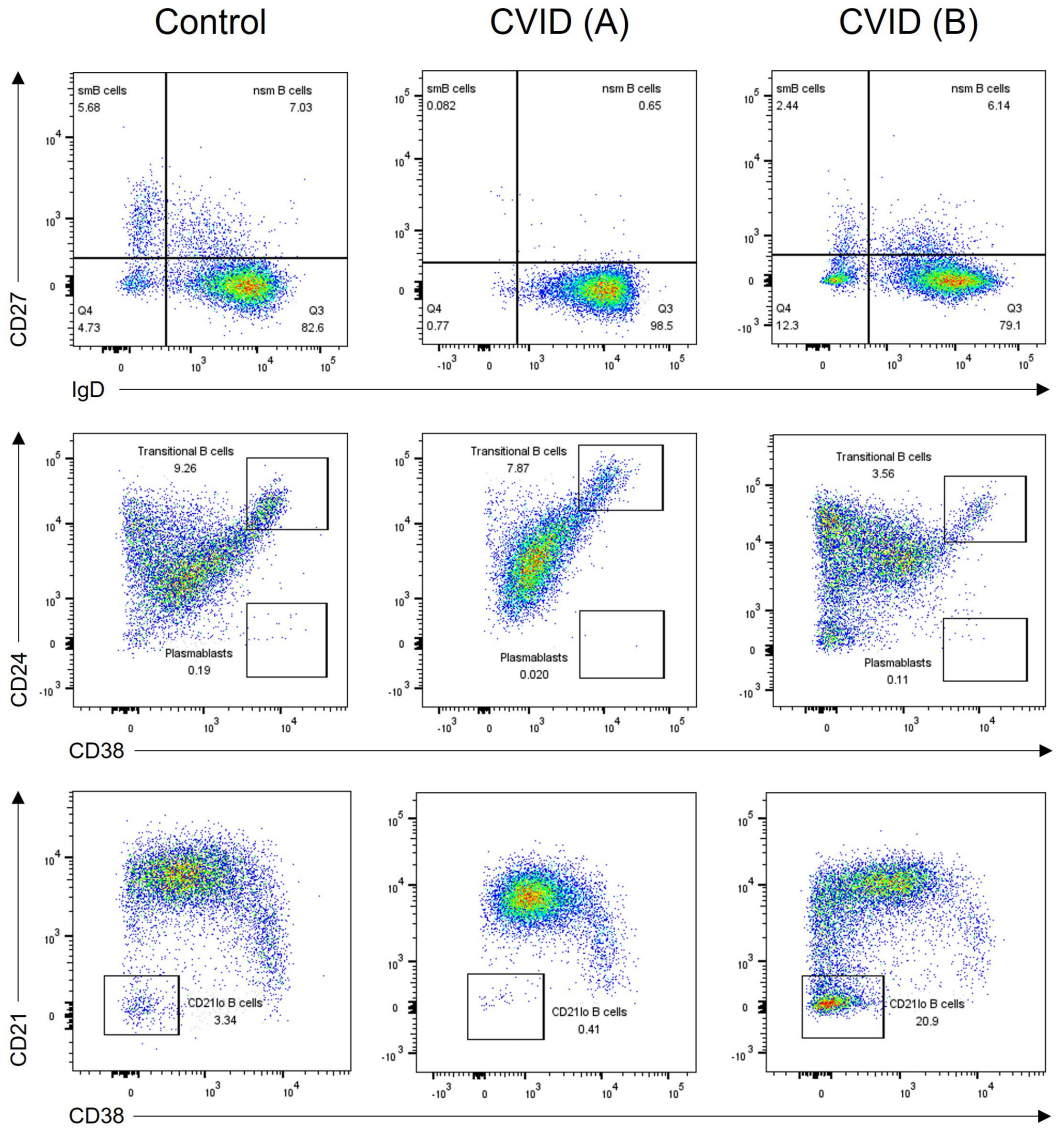
B-cell subpopulation analysis in two CVID patients and one healthy control. *CVID patient (A)* has reduced memory B-cell subsets including switched memory (CD27+sIgD-) and non-switched memory (CD27+sIgD+) B cells (middle panel). *CVID patient (B)* shows an expansion of CD21^lo^ B cells (right panel). CVID, common variable immunodeficiency; sm, switched memory; nsm, non-switched memory.

Seventy patients were diagnosed with CVID based on a comprehensive assessment of their clinical manifestations, immunoglobulin levels, and FCM analysis of B- and T-cell subsets. B-cell subpopulation analysis was performed in 63 patients demonstrating a significant reduction in switched memory B cells in 51 (80.9%) patients. Additionally, an expansion of transitional B cells and CD21^lo^ B cells was seen in 23.8% and 28.6% of patients, respectively (**Figure 4**). Genetic analysis through whole exome sequencing (WES) was carried out in three patients, revealing two cases of activated phosphoinositide 3 kinase (PI3K)-δ syndrome (APDS) caused by heterozygous GOF mutations in *PIK3CD*, and one TACI deficiency due to heterozygous mutation in *TNFRSF13B.* Both patients with APDS exhibited decreased naïve T cells, reduced number of switched memory B cells, and very high percentage of transitional B cells (**Table 2**).

### 3.5 Diseases of immune dysregulation

Forty-nine patients were diagnosed with a disorder of immune dysregulation. Among them, nine had CHS, seven had FHL, five had ALPS, two had autoimmune polyendocrinopathy candidiasis ectodermal dystrophy (APECED) and one had IPEX syndrome. Twenty patients (40.8%) received genetic testing. Biallelic germline pathogenic variants were identified in six different genes, i.e., *LYST*, *FAS*, *LRBA*, *UNC13D*, *RIPK1*, and *TPP2* (**Table 3**).

Elevated CD3+TCRαβ+CD4−CD8−T cells (>2.5% of CD3+ lymphocytes) was found in five unrelated patients with ALPS; two of them carried the same missense homozygous mutation (p. Phe133Val) in *FAS*. Increased TCRαβ DNT were also found in nine patients with other immunodeficiency disorders such as: CVID, STAT1-GOF, LRBA, ARPC1B, RIPK1, and SASH3 deficiencies. However, the expansion of TCRαβ DNT was more pronounced in patients with ALPS (ALPS: median= 22%, range: 8% – 67%; Other IEIs: median= 3.5%, range: 3% – 23%).

LRBA deficiency was diagnosed in three patients, all of whom presented with typical features, including autoimmune cytopenia, granulomatous-lymphocytic interstitial lung disease (GLILD), hypogammaglobulinemia, reduced switched memory B cells, and expanded CD21^lo^ B cells.

One patient out of the seven diagnosed with FHL exhibited a decreased perforin expression (as evidenced by a 10-fold reduction in MFI in comparison to control), despite maintaining a normal percentage of positive NK cells (97% of positivity) (**Figure 3**). Of the six remaining patients, five displayed defective/abnormal NK-cell degranulation. Moreover, two of three patients (85%) with CHS or GS2 had defective NK-cell degranulation (**Figure 3**).

All patients with RIPK1 deficiency presented with early-onset inflammatory bowel disease and decreased serum IgA and IgM levels. The FCM analysis unveiled reduced numbers of memory B cells and a concurrent elevation of transitional B cells in all cases.

### 3.6 Defects of phagocytes

Seven out of the 20 patients with phagocytic defects were diagnosed with CGD based on the FCM measurement of NADPH oxidase function. Notably, three male patients demonstrated a complete absence of respiratory burst, while four additional patients (2 males and 2 females) exhibited markedly reduced NADPH oxidase activity, indicated by a stimulation index (SI) ranging from 6 to 54 (**Table 2**).

### 3.7 Innate immunity defects

Four patients were diagnosed with MSMD; two of them had IL-12Rβ1 deficiency confirmed genetically. On patient had compound heterozygous mutations in *TYK2*, while the WES results weren’t conclusive for the last patient. Five patients with CMC displayed IL-17A production defect and hyperphosphorylation of STAT1 after IFN-γ stimulation. Heterozygote pathogenic variant in *STAT1* was identified in two patients (**Tables 2**, **3**).

### 3.8 Complement deficiencies

**Figure 5:**
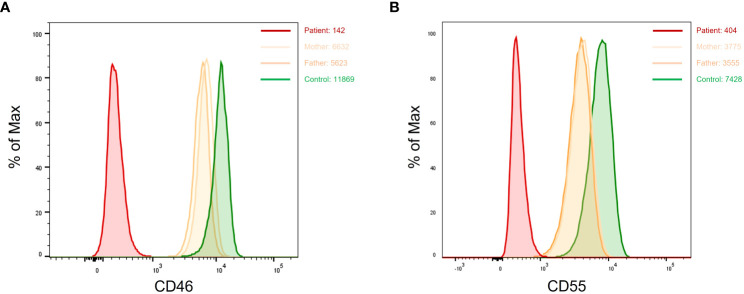
Dot plots showing complete loss of CD46 **(A)** and CD55 **(B)** expression in two patients with aHUS and CHAPLE disease, respectively. Carrier parents from both families show monomodal and intermediate expression of CD46 and CD55. aHUS, atypical hemolytic uremic syndrome; CHAPLE, complement hyperactivation angiopathic thrombosis and protein-losing enteropathy.

Patients with suspected aHUS were systematically screened for CD46 deficiency. Complete lack of CD46 expression (i.e., homozygous CD46 deficiency) was found in six patients, while partial defect of CD46 expression (likely heterozygous CD46 deficiency) was seen in one patient. Parents of patients with homozygous CD46 deficiency exhibited a heterozygous profile with a CD46 expression density representing ~ 50% of that of healthy controls. Based of FCM analysis, the diagnosis of homozygous CD55 (DAF) deficiency was established in six patients presenting with complement hyperactivation, angiopathic thrombosis, and protein-losing enteropathy (CHAPLE) disease. The assessment of CD55 expression in the family members of three patients unveiled an intermediate expression pattern, indicating a partial (heterozygous) defect in CD55 expression among the carrier parents (**Figure 5**).

## 4 Discussion

The department of medical biology at Rouiba hospital is one of the few centers in Algeria offering a comprehensive workup for patients with suspected IEI. In this study, we conducted a retrospective review of the use of FCM in the diagnosis of IEIs. Sharing insights into our practical experiences, we present FCM-based diagnostic approaches adapted to different clinical scenarios. In the past two decades, FCM has emerged as a highly valuable and versatile tool for diagnosing and studying IEIs (12). Individual FCM diagnostic testing depends on the patient’s clinical presentation and basic laboratory findings. The range of applications is broad, including phenotypic assays that investigate the numbers and percentages of immune cells (e.g., RTE, TCRαβ DNT, switched memory B cells), functional analysis of cellular processes (e.g., cytokine secretion, STAT phosphorylation, NK cell degranulation, NADPH oxidase activity in neutrophils), and direct analysis of potentially mutated cell membrane, and cytoplasmic proteins (**Table 1**) (9, 12, 19). The use of this diverse array of phenotypic and functional assays has proven instrumental in establishing definitive diagnoses for various disorders. However, it is important to note that these assays, if not conducted with meticulous attention to preanalytical and analytical considerations, may produce inaccurate results, potentially leading to misdiagnosis (12, 13, 20, 21). To implement such diagnostic assays with high and reproducible quality, a high level of expertise is required. Specifically, for protein expression and functional assays, in-house reference values need to be determined, and the parallel testing of healthy controls is highly recommended (9).

Over a nearly seven-year period, our laboratory diagnosed a total of 670 patients, with 70 different IEIs categorized into 9 different groups according to the IUIS classification (**Tables 2**, **3**). FCM has proven useful in diagnosing and categorizing IEI in 514 patients (76.7%). Based on our experience, FCM provided direct diagnostic insights for immunodeficiencies such as SCID, OS, MHC-II deficiency, WAS, XLA, XL-CGD, FHL, LAD-1, AR-CD46 deficiency, and CD55 deficiency. For certain IEIs, including HIES, STAT1-GOF, APDS, ALPS, IPEX, AR-CGD, and autosomal dominant (AD)-CD46 deficiency, FCM offered suggestive evidence, necessitating subsequent genetic testing for confirmation. FCM findings provided informative clues, although they lacked specificity for diagnosing hypomorphic V(D)J recombination defects, Ataxia Telangiectasia, CVID, LRBA deficiency, RIPK1 deficiency, and Good’s syndrome (**Table 4**). Additionally, in select IEIs such as selective IgA deficiency, transient hypogammaglobulinemia of infancy, congenital neutropenia, and early-onset inflammatory bowel disease, FCM played a crucial role in differential diagnosis and narrowing down possibilities.

**Table 4:** Flow cytometry-based diagnosis of IEIs.

IEI	Associated flow cytometry findings
Robust evidence	
SCID	T-cell count <300/μl
Omenn Syndrome	Absent or severely decreased naïve T cells and RTE
MHC-II deficiency	Absent or markedly decreased HLA-DR expression on B cells
WAS	Absent or markedly decreased WASp expression on T cells
XLA	Absent or very low (<2%) circulation B cells with absent/reduced Btk expression on monocytes
XL-CGD	Absent NADPH oxidase activity
FHL type 2	Absent or markedly decreased perforin expression on NK cells
FHL types 3, 4 and 5	Defective resting NK-cell degranulation assay
LAD-1	Absent CD18/CD11a expression on neutrophiles
AR-CD46 deficiency	Absent CD46 expression on T cells
CHAPLE disease	Absent CD55 expression on neutrophiles
Suggestive evidence	
CD3γ deficiency	Low CD3/TCRαβ expression on T cells
HIES	Markedly decreased Th17 cells or defective IL-17 production associated or not with STAT3 phosphorylation defect
CMC-STAT1 GOF	Markedly decreased Th17 cells or defective IL-17 production associated with hyperphosphorylation of STAT1
APDS	Expanded transitional B cells with reduced switched memory B cells and decreased naïve T cells
ALPS-FAS	Significant increase in TCRαβ DNT (>7% of CD3+ T cells)
IPEX syndrome	Absent or markedly decreased FOXP3^+^CD25^+^ Treg
AR-CGD	Reduced NADPH oxidase activity
AD-CD46 deficiency	~ 50% reduction of CD46 expression on lymphocytes
Informative clues	
Hypomorphic V(D)J recombination defects	Markedly decreased naïve T cells and RTE associated or not with reduced B cells
DiGeorge syndrome	Reduced naive T cells and RTE
Ataxia Telangiectasia	CD3 lymphopenia with reduced naive T cells
CVID	Markedly decreased switched memory B cells
LRBA deficiency	Reduced switched memory B cells with expanded CD21^lo^ B cells
RIPK1 deficiency	Decreased central and effector memory CD4 T cells Decreased switched and non-switched memory B cells Raised transitional B cells
Good’s syndrome	Absent or markedly decreased B cells Low CD4/CD8 ratio

AD, autosomal dominant; ALPS, autoimmune lymphoproliferative syndrome; APDS, activated PI3K delta syndrome; AR, autosomal recessive; CGD, chronic granulomatous disease; CHAPLE, complement hyperactivation angiopathic thrombosis and protein-losing enteropathy; CMC, chronic mucocutaneous candidiasis; CVID, common variable immunodeficiency; FHL, familial hemophagocytic lymphohistiocytosis; HIES, hyper-IgE syndrome; IPEX, immune dysregulation-polyendocrinopathy-enteropathy-x-linked; LRBA, LPS-responsive beige-like anchor protein; LAD, leukocyte adhesion deficiency; LRBA, LPS-responsive beige-like anchor protein; MHC, major histocompatibility complex; RIPK1, receptor-interacting serine/threonine-protein kinase 1; SCID, severe combined immunodeficiency; STAT, signal transducer and activator of transcription; WAS, Wiskott Aldrich syndrome; XLA, X-linked agammaglobulinemia.

**Figure 6:**
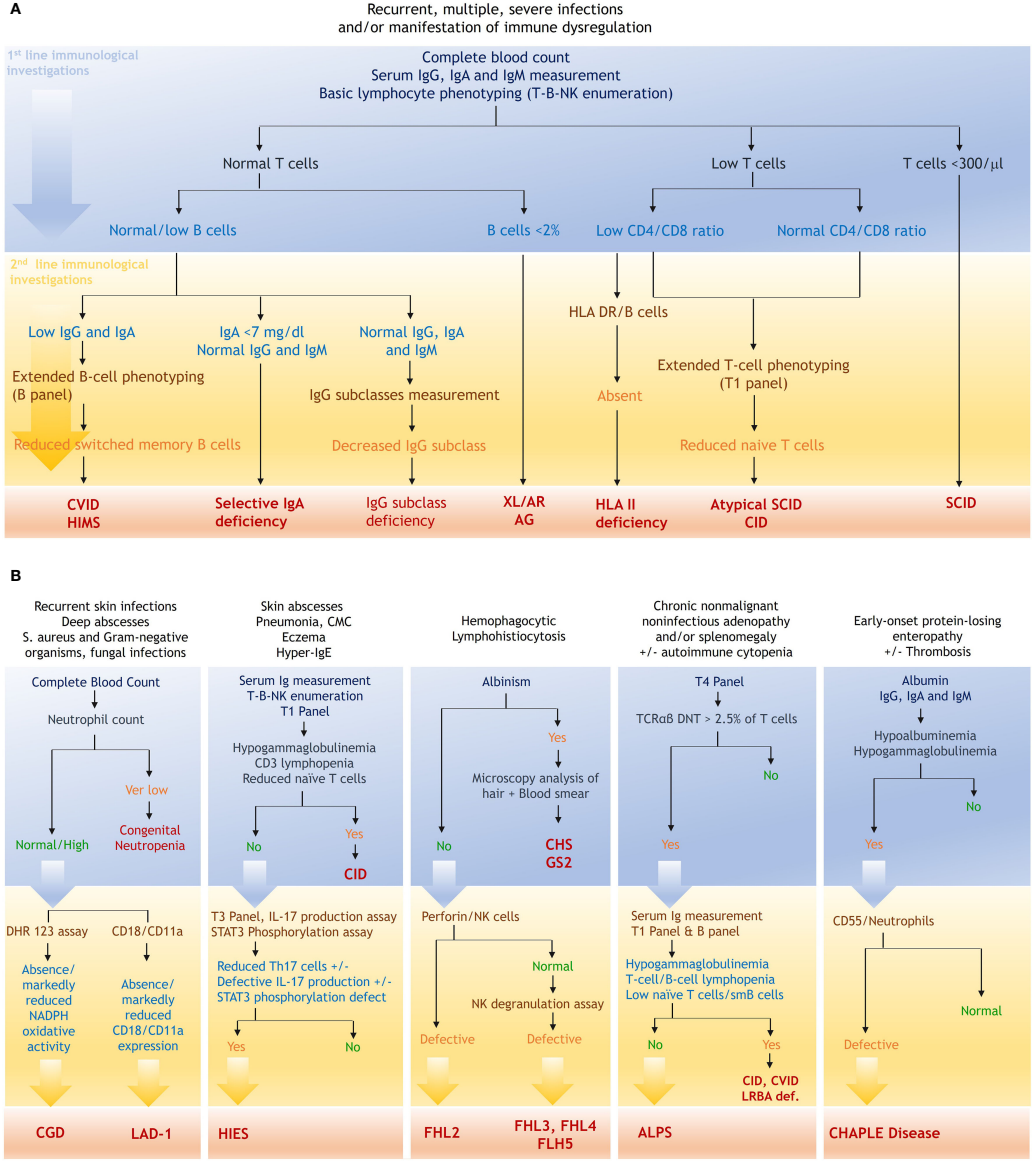
Flow cytometric workup based on the clinical presentation. **(A)**, FCM based strategy in the context of nonspecific IEI manifestations. **(B)**, diagnostic strategy in the context of specific clinical presentations. *T1 panel*, *CD3, CD4, CD8, CD45RA, CD45RO, CCR7; T3 panel*, *CD3, CD4, CD45RO, CXCR3, CCR6, CXCR5; T4 panel*, *TCRαβ, TCRγδ, CD3, CD4, CD8, CD45; B panel*, *CD19, CD27, sIgD, CD38, CD24, CD21*. ALPS, autoimmune lymphoproliferative syndrome; AR, autosomal recessive; CGD, chronic granulomatous disease; CHAPLE, complement hyperactivation angiopathic thrombosis and protein-losing enteropathy; CHS, Chediak-Higashi syndrome; CID, combined immunodeficiency; CVID, common variable immunodeficiency; DHR, Dihydrorhodamine; GS2, Griscelli syndrome type 2; HIES, hyper-IgE syndrome; HIMS, hyper-IgM syndrome; LAD, leukocyte adhesion deficiency; LRBA, LPS-responsive beige-like anchor protein; SCID, severe combined immunodeficiency; STAT, signal transducer and activator of transcription; XLA, X-linked agammaglobulinemia.

In our laboratory, the flow cytometric analysis is systematically structured according to the clinical scenario (**Figure 6**). For patients presenting with suspected CID, characterized by early onset severe/recurrent bacterial, fungal, and viral infections, along with severe reactions to live microorganism vaccines, the flow cytometric work-up starts with basic lymphocyte phenotyping. The identification of profound T lymphopenia (i.e., T cell count <300/μl) serves as a direct clue for the diagnosis of SCID (22). Simultaneous analysis of B and NK cell subpopulations aids in classifying SCID and facilitates the optimization of genetic testing through targeted Sanger sequencing. In our series, 30 out of the 163 patients (18.4%) diagnosed with combined immunodeficiency had SCID with a notable 70% exhibiting T-B-NK+ SCID. In contrast to patients with typical SCID, those harboring hypomorphic variants in SCID-causing genes typically manifest a less pronounced lymphopenia, thereby necessitating a more detailed analysis of naïve and memory subpopulations. Interestingly, despite substantial number of circulating T cells, naive T cells were absent in all patients with OS diagnosed in our laboratory (**Table 2**). Delving into the analysis of naive T cells, and more interestingly RTE in these patients, provides compelling evidence for an accurate diagnosis (**Figure 6**) (23). In addition to OS, leaky SCIDs form another category within the hypomorphic SCID spectrum. Patients with leaky SCID most often present with atypical phenotype characterized by less severe-but atypical-infections with a later onset, and a high frequency of immune dysregulation features, particularly autoimmune cytopenia (2, 24–26). T-B-NK enumeration in these patients often reveals slightly decreased to normal T-cell count, leading to a misdiagnosis (24). We previously reported a case of hypomorphic Artemis deficiency in a young adult, characterized by recurrent pneumonia, epidermodysplasia verruciformis, and a subtle T cell lymphopenia coupled with severe decrease in RTE (27). Given the diagnostic challenges inherent to such atypical entities, it appears reasonable to incorporate detailed T-cell phenotyping as a first-line investigation, optimizing diagnostic accuracy and minimizing the potential for misdiagnosis.

One hundred sixteen patients were diagnosed with *bona fide* CID. The most common immunophenotypic features were CD4 lymphopenia (78.4%), inverted CD4/CD8 ratio (64.7%), and reduced naïve CD4 (84.4%) and CD8 (87.8%) T cells. In addition to quantitative abnormalities, lymphocyte phenotyping can unveil subtle aberrations, such as low expression of CD3 complex on the T-cell surface. Indeed, we observed in two siblings, both affected with CD3γ deficiency, a 10-fold lower expression of the TCR-CD3 complex on the surface of T cells. Both brothers exhibited a clinical picture of CID with recurrent infections, autoimmune cytopenia, and lymphopenia. In view of these findings, FCM analysis should also carefully consider the intensity of CD3 expression.

MHC class II deficiency was the most prevalent CID in our series, accounting for 21.5% of all CIDs and 5.2% of total IEI cases. Genetic analysis conducted in 12 patients revealed pathogenic variants in the *RFXANK* gene in 10 patients, with 9 displaying an identical 26-base pair deletion (752delG26). The high frequency of the 752delG26 deletion in our patients is an accordance with earlier findings, underscoring a high occurrence of the same variant in over 90% of North African families (28, 29). This finding appears to be associated with a founder effect related to this mutation, as previously suggested by Ouederni et al. (30). Typically, the suspicion of MHC-II deficiency is raised in patients with recurrent infections, hypogammaglobulinemia, CD4 lymphopenia, and low CD4/CD8 ratio (31). However, it is noteworthy to mention that patients with MHC-II deficiency may paradoxically manifest a normal CD4+ T-cell count and serum immunoglobulin (Ig) levels. Notably, in our series, 11.4% of MHC-II deficient patients displayed a normal CD4+ T-cell count, 25.7% had normal CD4/CD8 ratio, and 45.7% maintained IgG concentration within the normal range (**Table 2**). Hence, it would be relevant to systematically screen patients of North African descent exhibiting a clinical presentation of CID for MHC-II deficiency, particularly in cases involving parental consanguinity, recurrent pneumonia, and chronic diarrhea.

Hyper IgE syndrome is a heterogeneous group of monogenic disorders characterized by the triad of high serum IgE levels, eczema, and recurrent skin and lung infections. AD-HIES caused by LOF mutations in *STAT3* gene is the prototype of these disorders. Additionally, six recently identified disorders (IL6ST, IL6R, ZNF341, ERBIN, CARD11, and TGFBR deficiencies) were included in the HIES spectrum, along with PGM3 and SPINK5 deficiencies (1, 32). Interestingly, high total IgE levels have also been reported in association with some CIDs, including DOCK8 deficiency, WAS, and ARPC1B deficiency (33–36). Accordantly, a crucial initial step in our approach for patients with suspected HIES is to rule out the presence of CID through a comprehensive T-cell phenotyping. Low Th17 cells and defective IL-17 production are characteristic features of patients with STAT3 deficiency and STAT3−related disorders, such as gp130, IL-6R, ZNF341, and PGM3 deficiencies (37–43). The analysis of CXCR3 and CCR6 markers on CD4+ memory T cells provides a straightforward approach for investigating Th17 lymphocytes (18). However, based on our experience, this phenotypic assay seems to be less sensitive in detecting impaired Th17 immunity compared to IL-17 production assay (**Table 2**). Furthermore, patients with certain disorders sharing clinical features with HIES, such as IL-21R deficiency, may exhibit diminished Th17 cells (44). The STAT3 phosphorylation assay, originally designed for STAT3 deficiency, does not consistently exhibit abnormalities in cases of STAT3 deficiency depending on the mutation’s location (45). Moreover, other STAT3-related HIES frequently manifest a defective STAT3 phosphorylation (39–43).

In the context of suspected primary antibody production defect, the flow cytometric work-up usually starts with T-B-NK enumeration (**Figure 6**). The absence or extremely low levels of B cells (<2% of circulating lymphocytes), accompanied by a significant reduction in total serum Ig levels and normal T cells strongly indicate a diagnosis of Agammaglobulinemia. Male patients with Agammaglobulinemia should be systematically screened for XLA-Btk deficiency. Since the introduction of the Btk expression assay in our laboratory, two patients have been tested and exhibited markedly decreased Btk expression in monocytes. According to the literature data, the majority of identified Btk mutations impair or abrogate Btk protein expression (9, 46). However, normal Btk protein levels do not exclude XLA, and in cases where clinical suspicion is high, genetic analysis should be performed (9, 15). Beyond the scope of Agammaglobulinemia, most patients with PAD display a less severe B lymphopenia, necessitating further B cell subset analysis (**Table 1**, **Figure 6**). Disturbances in B-cell subpopulations, such as reduced memory B cells as well as elevated transitional or CD21^lo^ B cells, provide direct insights into a primary origin for the antibody production defect, especially in adult patients where secondary causes of hypogammaglobulinemia are common (47).

Common variable immunodeficiency was the most prevalent PAD in our series, accounting for 45.2% of all PADs and 10.4% of total IEI cases. CVID is a heterogenous group of monogenic disorders characterized by reduced serum levels of IgG, IgA, and/or IgM, with impaired antibody response to both polysaccharide and protein antigens. Clinically, CVID patients suffer from an increased susceptibility to sinopulmonary infections along with a broad spectrum of inflammatory, granulomatous, and autoimmune diseases (48–50). Reduced numbers of switched memory B-cells, found in 80.9% of our CVID patients, represent the cellular hallmark of CVID and serve as a key diagnostic element according to ESID criteria (51, 52). Additional B-cell disturbances like expansion of transitional or CD21^lo^ B cells were also found in our CVID cohort. Genetic analysis through WES identified two patients with heterozygous GOF mutations in *PIK3CD*. Both patients presented with APDS symptoms (i.e., sinopulmonary infections, lymphoproliferation, enteropathy, and autoimmune hemolytic anemia), and displayed a characteristic immune profile including increased proportion of transitional B cells, reduced memory B cells, decreased naïve T cells, and elevated levels of serum IgM (53, 54).

A significant number of patients with CVID present some features of a cellular immunodeficiency and may, in fact, suffer from a certain form of CID (55). To exclude severe T-cell deficiency in patients with suspected CVID, we systematically conducted a comprehensive analysis of naïve and memory T-cell subpopulations. The revised criteria from the ESID registry require therefore a T-cell count of >200/μl with an amount of at least 10% of naïve CD4+CD45RA+ T cells in adults (52). von Spee-Mayer et al, reported that a reduction in naïve CD4 T cells to less than 10% had the highest sensitivity of all tested markers for patients with clinical complications often associated with CID, although the authors admitted that none of the current definitions sufficiently separates CID from CVID patients (56). It is noteworthy that both patients with APDS, as well as the one with TACI deficiency, exhibited a significant reduction in naïve CD4 T cells (less than 5%), despite being categorized as CVID.

Forty-nine patients were diagnosed with immune dysregulation disorders, including seven with FHL, five with ALPS, and three with LRBA deficiency. Elevated TCRαβ DNT represent a distinctive feature of ALPS and serve as a major diagnostic criterion (57). However, according to our experience, elevated TCRαβ DNT may also be found in other monogenic disorders, such as LRBA, ARPC1B, RIPK1, and SASH3 deficiencies, as well as CVID, and CMC with STAT1-GOF. Therefore, in addition to the clinical presentation, the extent of the expansion of TCRαβ DNT cells should be considered in the differential diagnosis, given that TCRαβ DNT cell elevation appears to be more pronounced in patients with ALPS. FCM analysis of patients with LRBA deficiency revealed a significant reduction in switched memory B cells and expanded CD21^lo^ B cells in all cases. While these B-cell disturbances are not specific, they offer valuable insights for diagnosing LRBA deficiency in patients with recurrent infections, features of immune dysregulation, and hypogammaglobulinemia (58).

Flow cytometric detection of perforin in NK cells has proven to be a rapid and sensitive test for identifying perforin deficiency (20). Typically, NK cells from patients with perforin deficiency exhibit either absent or markedly decreased perforin expression (20, 59). However, it is important to note that normal perforin expression does not definitively exclude defects in function or the presence of structurally abnormal proteins (20). In our laboratory, we have identified a patient displaying a perforin expression defect indicative of potential perforin deficiency. Remarkably, this investigated patient exhibited a strikingly normal percentage of positive NK cells (97%). However, he displayed a MFI that was notably tenfold lower compared to the control. Therefore, in the analysis of perforin expression, a comprehensive assessment should consider both the frequency and MFI for a more nuanced interpretation (60).

The DHR assay, measuring the NADPH oxidase activity, is a rapid and sensitive screening test for CGD (61). This assay can differentiate between X-CGD and AR-CGD (mainly caused by p47^phox^ defect) on the basis of distinctive DHR histogram SI and pattern (62–64). Vowells et al. demonstrated that the geometric mean SI from patients with CGD with defective gp91*^phox^* and p47*^phox^* were 1.3 (range, 0.9 to 2.2) and 13.2 (range, 3.5 to 52.1), respectively (61). In our cohort, three male patients displayed a complete lack of oxidative activity, indicating an XL form, while an additional four patients manifested a modest deviation in the DHR histogram with a SI ranging from 6 to 54, suggestive of a potential AR form. However, it is worth mentioning that this dichotomy is not consistently observed, as certain autosomal recessive forms, especially p22^phox^ deficiency, may present with null oxidase activity, and patients with confirmed X-CGD may exhibit a residual oxidase activity. In such instances, conducting the DHR assay for potential carriers (mothers) is crucial to identify mosaicism and predict the pattern of disease inheritance, although XL-CGD cases due to *de novo* mutation have been reported (65, 66). Additionally, further flow cytometric analysis of NADPH oxidase enzyme subunits (i.e., gp91^phox^, p22^phox^, p47^phox^, and p67^phox^) in patients and carrier mothers is useful in predicting the defective gene and enabling targeted genetic sequencing, thereby allowing for a rapid and cost-effective diagnosis of CGD (66).

Based on our experience, FCM analysis has proved to be effective in diagnosing CD46 and CD55 deficiencies. Among patients with aHUS, seven exhibited either absent or significantly decreased CD46 expression suggesting both AR and AD forms of the disease. In addition, six patients presenting with PLE displayed complete loss of CD55 expression, all of whom carried biallelic mutations in the CD55 gene. In the context of surface complement protein detection, both percentages and MFI of CD46/CD55 expression were analyzed. The reference MFI values were established from a group of 10 healthy subjects. Homozygous-deficient patients exhibited MFIs comparable to the isotypic control, whereas those with heterozygous deficiency demonstrated an MFI approximately 50% of the mean MFI of the control group. Unlike some X-linked disorders such as WAS, XLA, and X-linked HIMS, CD46/CD55 heterozygous carriers did not display a bimodal expression pattern on their leukocytes. Rather, they exhibited monomodal distribution with intermediate fluorescence intensity compared with healthy controls (67–69) (**Figure 5**).

Over the past 7 years, our laboratory has enthusiastically embraced a diverse array of phenotyping and functional assays. While significant progress has been made, we recognize that there remain areas where our testing capabilities can be further enhanced. Therefore, we are dedicated to enhancing our testing panel by introducing new phenotypic and functional assays. Specifically, we aim to incorporate new markers such as TCRα signature (TCR Vα7.2) in V(D)J recombination defects, CD57 as a marker of senescent lymphocytes in APDS, gp91^phox^, p22^phox^, p47^phox^, and p67^phox^ in CGD, as well as signaling lymphocyte activation molecule (SLAM)–associated protein (SAP) and X-linked inhibitor of apoptosis protein (XIAP) in X-linked lymphoproliferative diseases 1 and 2 (12, 66, 70, 71). Additionally, we aspire to integrate lymphocyte proliferation assays into routine practice and develop new functional tests, such as the assessing of histone H2AX phosphorylation (γH2AX) in CD3 T cells following irradiation, commonly used in diagnosing Ataxia Telangiectasia and radiosensitive SCIDs (72, 73).

## 5 Conclusion

Flow cytometry has emerged as a highly valuable and cost-effective tool for diagnosing and studying IEIs, particularly in low-income countries where access to genetic testing is limited. A stratified flow cytometric analysis of specific proteins or particular subpopulations plays a crucial role in establishing definitive diagnoses for various disorders. While molecular testing remains necessary for a definitive diagnosis in some IEI disorders, conducting phenotypic or functional analyses of lymphocyte subsets provides clues to the underlying genetic defects or aids in narrowing down the list of putative genes to be analyzed.

## Data availability statement

The datasets presented in this study can be found in online repositories. The names of the repository/repositories and accession number(s) can be found below: PRJNA1089953 (SRA).

## Ethics statement

The studies involving humans were approved by Ethics committee of Rouiba Hospital. The studies were conducted in accordance with the local legislation and institutional requirements. Written informed consent for participation in this study was provided by the participants’ legal guardians/next of kin.

## Author contributions

AT: Conceptualization, Data curation, Formal analysis, Funding acquisition, Investigation, Methodology, Resources, Validation, Writing – original draft. RBe: Data curation, Writing – review & editing. AY: Data curation, Investigation, Writing – review & editing. SH: Data curation, Writing – review & editing. FF: Data curation, Writing – review & editing. MK: Data curation, Writing – review & editing. HBe: Data curation, Writing – review & editing. ST: Data curation, Writing – review & editing. SA: Data curation, Writing – review & editing. JS: Data curation, Writing – review & editing. JN: Data curation, Writing – review & editing. FZ: Data curation, Writing – review & editing. SM: Data curation, Writing – review & editing. RA: Writing – review & editing. AS: Writing – review & editing. YF: Writing – review & editing. AK: Writing – review & editing. HMe: Writing – review & editing. TB: Data curation, Writing – review & editing. ZBe: Data curation, Writing – review & editing. AD: Writing – review & editing. KO: Writing – review & editing. FA: Data curation, Investigation, Writing – review & editing. MF: Writing – review & editing. CBe: Data curation, Writing – review & editing. RK: Data curation, Writing – review & editing. AO: Data curation, Writing – review & editing. AS: Writing – review & editing. IB: Writing – review & editing. CBo: Writing – review & editing. NBo: Writing – review & editing. IM: Writing – review & editing. NK: Writing – review & editing. HBo: Software, Writing – review & editing. TK: Writing – review & editing. FM: Writing – review & editing. MBou: Writing – review & editing. AZ: Writing – review & editing. OG: Writing – review & editing. ML: Writing – review & editing. AM: Writing – review & editing. NBe: Writing – review & editing. MBen: Writing – review & editing. ZZ: Writing – review & editing. BB: Data curation, Investigation, Writing – review & editing. MuB: Writing – review & editing. BA: Writing – review & editing. ZBo: Writing – review & editing. OI: Writing – review & editing. HMa: Data curation, Investigation, Writing – review & editing. LK: Data curation, Investigation, Writing – review & editing. LS: Writing – review & editing. RBouk: Supervision, Writing – review & editing. CL: Data curation, Investigation, Writing – review & editing. SR: Data curation, Investigation, Writing – review & editing. LN: Data curation, Investigation, Writing – review & editing. KD: Data curation, Investigation, Writing – review & editing.

## Supplementary material

Supplementary Figure 1 IEI distribution in our series. ALPS, autoimmune lymphoproliferative syndrome; APDS, activated PI3K delta syndrome; ARPC1B, actin related protein 2/3 complex subunit 1B; CGD, chronic granulomatous disease; CID, combined immunodeficiency; CHAPLE, complement hyperactivation angiopathic thrombosis and protein-losing enteropathy; CMC, chronic mucocutaneous candidiasis; CVID, common variable immunodeficiency; fHLH, familial hemophagocytic lymphohistiocytosis; HIES, hyper-IgE syndrome; IPEX, immune dysregulation-polyendocrinopathy-enteropathy-x-linked; LAD, leukocyte adhesion deficiency; LRBA, LPS-responsive beige-like anchor protein; MHC, major histocompatibility complex; MSMD, mendelian susceptibility to mycobacterial disease, OS, Omenn syndrome; PAD, predominantly antibody deficiencies; RIPK1, receptor-interacting serine/threonine-protein kinase 1; SCID, severe combined immunodeficiency; SIAD, selective IgA deficiency; TPP2, tripeptidyl peptidase 2; STAT, signal transducer and activator of transcription.
