# Supplementary figures and images for: Flow cytometry-based diagnostic approach for inborn errors of immunity: experience from Algeria
Source: Front Immunol. 2024 Jul 12;15:1402038. doi: 10.3389/fimmu.2024.1402038 (PMC11273131; doi:10.3389/fimmu.2024.1402038)

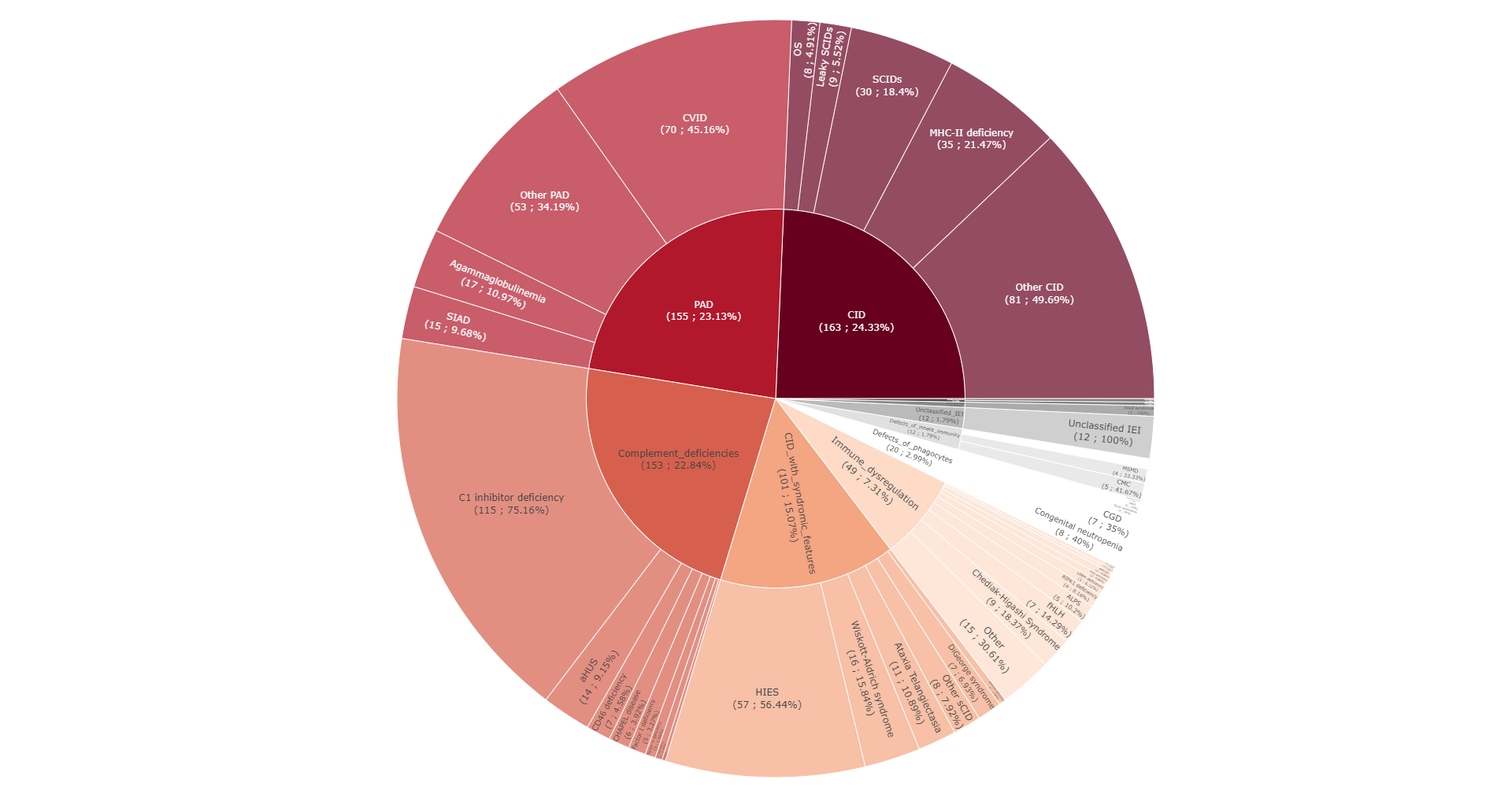

Supplement: Supplementary Figure 1 — IEI distribution in our series. ALPS, autoimmune lymphoproliferative syndrome; APDS, activated PI3K delta syndrome; ARPC1B, actin related protein 2/3 complex subunit 1B; CGD, chronic granulomatous disease; CID, combined immunodeficiency; CHAPLE, complement hyperactivation angiopathic thrombosis and protein-losing enteropathy; CMC, chronic mucocutaneous candidiasis; CVID, common variable immunodeficiency; fHLH, familial hemophagocytic lymphohistiocytosis; HIES, hyper-IgE syndrome; IPEX, immune dysregulation-polyendocrinopathy-enteropathy-x-linked; LAD, leukocyte adhesion deficiency; LRBA, LPS-responsive beige-like anchor protein; MHC, major histocompatibility complex; MSMD, mendelian susceptibility to mycobacterial disease, OS, Omenn syndrome; PAD, predominantly antibody deficiencies; RIPK1, receptor-interacting serine/threonine-protein kinase 1; SCID, severe combined immunodeficiency; SIAD, selective IgA deficiency; TPP2, tripeptidyl peptidase 2; STAT, signal transducer and activator of transcription. [file Image_1.png]
