# Supplementary material for: Flow cytometry-based diagnostic approach for inborn errors of immunity: experience from Algeria
Source: Front Immunol. 2024 Jul 12;15:1402038. doi: 10.3389/fimmu.2024.1402038 (PMC11273131; doi:10.3389/fimmu.2024.1402038)
Supplement: Supplementary file 2 [file DataSheet_1.docx]

**SUPPLEMENTARY METHODS**

**Analysis of surface CD132 (common** γ **chain) expression**

1. Whole blood samples from the patient and healthy control are stained with CD3-FITC (Clone: SK7, BD Biosciences), CD19-PECy7 (Clone: SJ25C1, BD Biosciences), CD45-APCH7 (Clone: 2D1, BD Biosciences), and CD132-PE (Clone: AG184, BD Pharmingen) or PE-conjugated IgG1, κ isotype control (Clone: MOPC-21, BD Pharmingen) at room temperature (RT) for 20 minutes (min) in the dark.
2. Lyse erythrocytes using 1X FACS Lysing Solution (BD Biosciences: 349202).
3. Wash cells with FACS washing buffer (PBS with 1% FBS).
4. Stained cells are analyzed by flow cytometer. The analysis gate is set for B cells.

**Analysis of surface CD127 (IL-7RA) expression**

1. Whole blood samples from the patient and healthy control are stained with CD3-APC (Clone: SK7, BD Biosciences), CD45-APCH7 (Clone: 2D1, BD Biosciences), and CD127-PE (Clone: HIL-7R-M21, BD Pharmingen) or PE-conjugated IgG1, κ isotype control (Clone: MOPC-21, BD Pharmingen) at RT for 20 min in the dark.
2. Lyse erythrocytes using 1X FACS Lysing Solution.
3. Wash cells with FACS washing buffer.
4. Stained cells are analyzed by FACS.

**Analysis of surface HLA-DR expression**

1. Whole blood samples are stained with CD19-FITC (Clone: 4G7, BD Biosciences), CD3-PerCP Cy5.5 (Clone: SK7, BD Biosciences), CD14-PECy7 (Clone: M5E2, BD Biosciences), CD45-APCH7 (Clone: 2D1, BD Biosciences), and HLA-DR-PE (Clone: L243) at RT for 20 min in the dark.
2. Lyse erythrocytes using 1X FACS Lysing Solution.
3. Wash cells with FACS washing buffer.
4. Stained cells are analyzed by flow cytometer. The analysis gate is set for B cells and monocytes.

**Analysis of surface CD46/MCP expression**

1. Whole blood samples are stained with CD3-APC (SK7, BD Biosciences), CD45-APCH7 (Clone: 2D1, BD Biosciences), and CD46-PE (Clone: E4.3, BD Pharmingen) or PE-conjugated IgG2a, κ isotype control (Clone: G155-178, BD Pharmingen) at RT for 20 min in the dark.
2. Lyse erythrocytes using 1X FACS Lysing Solution.
3. Wash cells with FACS washing buffer.
4. Stained cells are analyzed by flow cytometer. Acquire 10,000 events in a gate set on T cells. Lymphocytes are gated using CD45 *vs.* side scatter (SSC) and T cells based on CD3 expression. Both percentage and MFI of CD46 expression are determined and compared to controls.

**Analysis of surface CD55/DAF expression**

1. Whole blood samples are stained with CD15-APC (Clone: HI98, BD Pharmingen), CD45-APCH7 (Clone: 2D1, BD Biosciences), and CD55-PE (Clone: IA10, BD Biosciences) or PE-conjugated IgG2a, κ isotype control (Clone: G155-178, BD Pharmingen) at RT for 20 min in the dark.
2. Lyse erythrocytes using 1X FACS Lysing Solution.
3. Wash cells with FACS washing buffer.
4. Stained cells are analyzed by flow cytometer. Acquire 10,000 events in a gate set on neutrophils using SSC/CD45 –> SSC/CD15 gating strategy. Both percentage and MFI of CD55 expression are determined and compared to controls.

**Protocol for intracellular WASp staining**

1. Whole blood samples from the patient and healthy control are stained with CD3-PE (Clone: SK7, BD Biosciences) and CD45-APCH7 (Clone: 2D1, BD Biosciences) for 20 min at room temperature in the dark.
2. Lyse erythrocytes using 1X FACS Lysing Solution.
3. Wash cells with FACS Staining Buffer (BD Pharmingen: 554656), then centrifuge and remove the supernatant.
4. Add 500μL of Fixation/Permeabilization solution (BD: 555028).
5. Incubate the cells for 20 min at RT.
6. Wash the cells.
7. Add 2mL of Perm/Wash buffer (BD: 555028).
8. Incubate the cells for 10 min at RT.
9. Wash the cells and re-suspended in 100 μL Perm/Wash buffer.
10. Add anti-WASp (Clone: 5A5, BD Pharmingen) in the first tube and IgG2a, κ isotype control (Clone: G155-178, BD Pharmingen) in the second one.
11. Incubate the cells for 30 min at room temperature.
12. Wash the cells.
13. Add secondary antibody-FITC (Clone: R19-15, BD Pharmingen).
14. Incubate the cells for 30 min at RT.
15. Wash the cells.
16. Stained cells are analyzed by flow cytometer. The analysis gate is set for T cells.

**Protocol for intracellular ZAP70 staining**

1. Whole blood samples from the patient and healthy control are stained with CD3-FITC (Clone: SK7, BD Biosciences), CD8-PECy7 (Clone: SK1, BD Biosciences), CD4-APCH7 (Clone: SK3, BD Biosciences), and CD45-V500 (Clone: 2D1, BD Biosciences), at RT for 20 min in the dark.
2. Red blood cells are lysed using 1X FACS Lysing Solution.
3. Cells are permeabilized with 1X BD FACS Permeabilizing Solution 2 at RT for 15 minutes.
4. Wash cells with FACS Staining Buffer and re-suspended in 100 μL.
5. Cells are stained with PE-conjugated anti-ZAP70 (Clone: 1E7.2, BD Biosciences) or IgG1, κ isotype control (Clone: MOPC-21, BD Pharmingen) at RT for 20 min in the dark.
6. Wash the cells.
7. Stained cells are analyzed by flow cytometer. The analysis gate is set for T cells.

**Protocol for intracellular BTK staining**

1. Whole blood samples from the patient and healthy control are stained with CD14-PECy7 (Clone: M5E2, BD Biosciences), and CD45-V500 (Clone: 2D1, BD Biosciences), at RT for 20 min in the dark.
2. Red blood cells are lysed using 1X FACS Lysing Solution.
3. Cells are permeabilized with 1X BD FACS Permeabilizing Solution 2 at RT for 15 minutes.
4. Wash cells with FACS Staining Buffer and re-suspended in 100 μL.
5. Cells are stained with PE-conjugated anti-BTK (Clone: 53/BTK, BD Biosciences) or IgG2a, κ isotype control (Clone: G155-178, BD Biosciences) at RT for 20 min in the dark.
6. Wash the cells.
7. Stained cells are analyzed by flow cytometer. The analysis gate is set for monocytes by side scatter and CD14 expression.

**Protocol for intracellular Perforin staining**

1. Whole blood samples are stained with CD3-FITC (Clone: SK7, BD Biosciences), and CD56-APC (NCAM16.2, BD Biosciences), at room temperature for 20 min in the dark.
2. Lyse red blood cells using 1X FACS Lysing Solution.
3. Cells are permeabilized with 1X BD FACS Permeabilizing Solution 2 at RT for 15 minutes.
4. Wash cells with FACS Staining Buffer and re-suspended in 100 μL.
5. Cells are stained with PE-conjugated anti-Perforin (Clone: δG9, BD Pharmingen) or IgG2b, κ isotype control (Clone: 27-35, BD Pharmingen) at RT for 20 min in the dark.
6. Wash cells with washing buffer.
7. Stained cells are analyzed by flow cytometer. The analysis gate is set for NK cells by CD3 and CD56 expression (NK cells are: CD56^+^CD3^−^).

**Analysis of surface CD40L (CD154) expression**

1. Peripheral blood mononuclear cells (PBMC) (1x10^6^) from the patient and healthy control are stimulated with PMA (20ng/mL) (Sigma-Aldrich: P8139) and ionomycin (1μg/mL) (Sigma-Aldrich: I0634) for 4 hours at 37°C. An untreated control sample should be set up in parallel.
2. Wash the cells and re-suspended in 100 μL of FACS Staining Buffer.
3. Add CD154-PE (Clone: 89-76, BD Biosciences) with additional surface markers including CD3-APC (Clone: SK7, BD Biosciences), CD8-PECy7 (Clone: SK1, BD Biosciences), and CD69-FITC (Clone: L78, BD Biosciences).
4. Incubate the cells for 20 min at RT in the dark.
5. Wash the cells.
6. Stained cells were analyzed by FACS. Compare expressions of CD154 of CD3+CD8- cells.

**STAT1 phosphorylation assay**

1. PBMC (1x10^6^) from the patient and healthy control are stimulated with 1,000 IU of recombinant human IFN-γ or with 50,000 IU of IFN-α for 20 min at 37°C in serum-free RPMI1680. An untreated control sample should be set up in parallel.
2. Fix the cells immediately by adding an equal volume of pre-warmed BD Cytofix Buffer (BD Biosciences: 554655). Mix well and incubate the tubes at 37°C for 10 min. Spin down the cells at 600g for 7 minutes.
3. Wash cells with FACS Staining Buffer.
4. Permeabilize the cells with BD Phosflow Perm Buffer III (BD Biosciences: 558050). Incubate on ice for 30 minutes and then spin down the cells.
5. Wash the cells and re-suspended in 100 μL of FACS Staining Buffer.
6. Add pSTAT1-Alexa Fluor 647 (pY701) (Clone: 4a, BD Biosciences) with monocytes and lymphocyte surface markers including CD14-BV421 (Clone: MφP9, BD Biosciences), CD3-PE (Clone: SK7, BD Biosciences), and CD4-FITC (Clone: RPA-T4, BD Biosciences).
7. Incubate cells for 30 min at 4°C.
8. Wash the cells twice.
9. Stained cells are analyzed by FACS. A stimulation index (SI) is calculated for gated monocytes/CD4+ (SI: the ratio of the MFI of the stimulated to the unstimulated cells).

**STAT3 phosphorylation assay**

1. PBMC (1x10^6^) from the patient and healthy control are stimulated with recombinant human IL-6 (0.1 μg/mL) (BD Pharmingen: 550071) for 15 min at 37°C. An untreated control sample should be set up in parallel.
2. Fix the cells immediately by adding an equal volume of pre-warmed BD Cytofix Buffer (BD Biosciences: 554655). Mix well and incubate the tubes at 37°C for 10 min. Spin down the cells at 600g for 7 minutes.
3. Wash cells with FACS Staining Buffer.
4. Permeabilize the cells with BD Phosflow Perm Buffer III (BD Biosciences: 558050). Incubate on ice for 30 minutes and then spin down the cells.
5. Wash the cells and re-suspended in 100 μL of FACS Staining Buffer.
6. Add pSTAT3-PE (pY705) (Clone: 4/P-STAT3, BD Biosciences) with lymphocyte surface markers including CD3-PerCP-Cy5.5 (Clone: SK7, BD Biosciences), and CD4-APC (Clone: RPA-T4, BD Biosciences).
7. Incubate cells for 30 min at 4°C.
8. Wash the cells twice.
9. Stained cells are analyzed by FACS. A SI is calculated for gated CD4+ T cells (SI: the ratio of the MFI of the stimulated to the unstimulated cells).

**IL-17A/** **IFN-γ/IL-4 production assay**

1. PBMC are suspended at a density of 1x10^6^ cells/mL.
2. Add 2 µL of Leukocyte Activation Cocktail (PMA: 40.5 µM + Ionomycin: 669.3 µM + Brefeldin A (protein transport inhibitor): 2.5 mg/ml) (BD Pharmingen: 550583) to 1 mL of cell suspension and mix thoroughly. Place culture in a 37°C humidified CO2 incubator for 5 hours. Untreated control sample should be set up in parallel.
3. Harvest and wash cells with FACS Staining Buffer.
4. Fix the cells by adding 1 mL of BD Cytofix Buffer (BD Biosciences: 554655). Mix well and incubate the tubes at 37°C for 10 min. Spin down the cells at 300g for 10 minutes.
5. Wash cells with FACS Staining Buffer.
6. Permeabilize the cells with 1x BD Perm/Wash buffer (BD Biosciences: 554723). Incubate at RT for 15 minutes and then spin down the cells.
7. Resuspend fixed/permeabilized cells in 50 μL of 1x BD Perm/Wash buffer and add antibodies cocktail: CD4 PerCP-Cy5.5 (clone: SK3, BD Pharmingen), IL-17A PE (clone: N49-653, BD Pharmingen), IFN-γ FITC (clone: B27, BD Pharmingen), IL-4 APC (clone: MP4-25D2, BD Pharmingen). Incubate at RT for 30 minutes in the dark.
8. Wash cells twice with 1mL of 1× BD Perm/Wash™ buffer and suspend in Stain Buffer prior to flow-cytometric analysis. Acquire at least 20,000 to 30,000 CD4+ lymphocytes.

**Degranulation assay of resting NK cells**

1. For each patient/control, 2 tubes are processed, i.e., unstimulated, and PMA + Ionomycin stimulated.
2. Add 5 𝜇L of FITC conjugated anti-CD107a (Clone: H4A3, BD Pharmingen) to 100 𝜇L of whole blood sample (ALC ≈ 2 × 10^5^ cells/mL).
3. For the stimulated tube, add 100 𝜇L of PMA (0.15 𝜇g/mL) and 100 𝜇L of Ionomycin (3 𝜇g/mL) and adjust the final volume to 500 𝜇L with incomplete RPMI 1640 media.
4. Incubate tubes for 2 hours at 37°C under 5% CO2. Unstimulated samples are incubated without stimulants to detect spontaneous degranulation.
5. Wash samples with FACS Staining Buffer and stain with CD56-PE (NCAM16.2, BD Biosciences), CD3-APC (Clone: SK7, BD Biosciences), and CD45-V500 (Clone: 2D1, BD Biosciences).
6. Lyse erythrocytes using 1X FACS Lysing Solution.
7. Wash cells with washing buffer.
8. Stained cells are analyzed by FACS. Lymphocytes are gated using CD45 *vs.* SSC and NK cells based on CD56+CD3−. The cut-off is set on unstimulated tubes and the increased CD107a expression in the same sample after stimulation is evaluated. ΔCD107a, which is the difference of surface CD107a expression between stimulated and non-stimulated NK cells, is determined.

**Dihydrorhodamine 123-based detection of reactive oxygen species in granulocytes (Dihydrorhodamine (DHR) 123 assay)**

1. Transfer 100 μL of heparinized blood per stimulation condition into a corresponding FACS tube: 20 μL of NaCl (0.9%) in the first tube (negative control) and 20 μL of PMA (8.1 μM) (Sigma-Aldrich) to the second tube.
2. Add 20 μL of DHR-123 solution to each tube (10 μg/mL) (Sigma-Aldrich: D1054) and vortex thoroughly.
3. Incubate all tubes for 20 min in a water bath at 37°C (protect from light).
4. Lyse erythrocytes with 1X FACS Lysing Solution.
5. Wash cells with washing buffer.
6. Production of reactive oxygen species of granulocytes is quantified by measuring intracellular rhodamine using FACS. Acquire 25,000 events in a gate set on granulocytes using a FCS/SSC-dot plot. A SI is calculated (SI: the ratio of the MFI of the stimulated to the unstimulated granulocytes).
