# Supplementary material for: Flow cytometry-based diagnostic approach for inborn errors of immunity: experience from Algeria
Source: Front Immunol. 2024 Jul 12;15:1402038. doi: 10.3389/fimmu.2024.1402038 (PMC11273131; doi:10.3389/fimmu.2024.1402038)
Supplement: Supplementary file 3 [file Table_1.docx]

**Supplementary Table S1:** Multicolor analysis of T-, B- and NK-cell subpopulations for characterization of IEI

| Antigen/Marker | Fluorochrome | Clone | Source | Ab/100 μl blood |
| --- | --- | --- | --- | --- |
| **T-B-NK Panel:** general lymphocyte overview | | | | |
| CD3 | FITC | SK7 | BD Biosciences | 5 μl |
| CD16/56 | PE | B73.1/NCAM16.2 | BD Biosciences | 5 μl |
| CD45 | PerCP Cy5.5 | 2D1 | BD Biosciences | 5 μl |
| CD4 | PE-Cy7 | SK3 | BD Biosciences | 5 μl |
| CD19 | APC | SJ25C1 | BD Biosciences | 5 μl |
| CD8 | APC-H7 | SK1 | BD Biosciences | 5 μl |
| **T1 panel:** naive and memory T-cell subsets | | | | |
| CD8 | FITC | SK1 | BD Biosciences | 4 μl |
| CCR7 | PE | 3D12 | BD Pharmingen | 10 μl |
| CD3 | PerCP Cy5.5 | SK7 | BD Biosciences | 6 μl |
| CD45RA | PE-Cy7 | HI100 | BD Pharmingen | 4 μl |
| CD45RO | APC | UCHL1 | BD Biosciences | 4 μl |
| CD4 | APC-H7 | SK3 | BD Biosciences | 4 μl |
| **T2 panel:** recent thymic emigrants | | | | |
| CD8 | FITC | SK1 | BD Biosciences | 4 μl |
| CD31 | PE | WM59 | BD Pharmingen | 20 μl |
| CD3 | PerCP Cy5.5 | SK7 | BD Biosciences | 6 μl |
| CD45RA | PE-Cy7 | HI100 | BD Pharmingen | 4 μl |
| CD45RO | APC | UCHL1 | BD Biosciences | 4 μl |
| CD4 | APC-H7 | SK3 | BD Biosciences | 4 μl |
| **T3 panel:** CD4+ memory T-cell subsets | | | | |
| CD3 | FITC | SK7 | BD Biosciences | 6 μl |
| CCR6 | PE | 11A9 | BD Pharmingen | 6 μl |
| CXCR5 | PerCP Cy5.5 | RF8B2 | BD Pharmingen | 4 μl |
| CD45RO | PE-Cy7 | UCHL1 | BD Biosciences | 4 μl |
| CXCR3 | APC | 1C6/CXCR3 | BD Pharmingen | 6 μl |
| CD4 | APC-H7 | SK3 | BD Biosciences | 4 μl |
| **T4 panel:** TCRαβ and TCRγδ T-cell subsets | | | | |
| TCRαβ | FITC | WT31 | BD Biosciences | 6 μl |
| TCRγδ | PE | 11F2 | BD Biosciences | 6 μl |
| CD3 | PerCP Cy5.5 | SK7 | BD Biosciences | 6 μl |
| CD8 | PE-Cy7 | SK1 | BD Biosciences | 4 μl |
| CD4 | APC | SK3 | BD Biosciences | 4 μl |
| CD45 | APC-H7 | 2D1 | BD Biosciences | 5 μl |
| **T5 panel:** regulatory T cells | | | | |
| CD3 | FITC | SK7 | BD Biosciences | 6 μl |
| CD25 | PE | M-A251 | BD Biosciences | 20 μl |
| / | PerCP Cy5.5 | / | / | / |
| / | PE-Cy7 | / | / | / |
| FoxP3 | Alexa Fluor® 647 | 259D/C7 | BD Pharmingen | 20 μl |
| CD4 | APC-H7 | SK3 | BD Biosciences | 4 μl |
| **T5bis panel:** regulatory T cells | | | | |
| CD3 | FITC | SK7 | BD Biosciences | 6 μl |
| CD127 | PE | HIL-7R-M21 | BD Pharmingen | 20 μl |
| CD8 | PerCP Cy5.5 | SK1 | BD Pharmingen | 5 μl |
| CD45RA | PE-Cy7 | UCHL1 | BD Biosciences | 4 μl |
| CD25 | APC | M-A251 | BD Pharmingen | 20 μl |
| CD4 | APC-H7 | SK3 | BD Biosciences | 4 μl |
| **B panel:** B-cell subsets | | | | |
| IgD | FITC | IA6-2 | BD Pharmingen | 10 μl |
| CD21 | PE | B-ly4 | BD Pharmingen | 10 μl |
| CD27 | PerCP Cy5.5 | L128 | BD Biosciences | 10 μl |
| CD24 | PE-Cy7 | ML5 | BD Pharmingen | 5 μl |
| CD38 | APC | HB7 | BD Pharmingen | 5 μl |
| CD19 | APC-H7 | SJ25C1 | BD Biosciences | 10 μl |
