# Supplementary material for: Flow cytometry-based diagnostic approach for inborn errors of immunity: experience from Algeria
Source: Front Immunol. 2024 Jul 12;15:1402038. doi: 10.3389/fimmu.2024.1402038 (PMC11273131; doi:10.3389/fimmu.2024.1402038)
Supplement: Supplementary file 4 [file Table_2.docx]

**Supplementary Table S2:** Flow cytometry-based diagnosis within IUIS categories.

| Category | Flow cytometry-based diagnosis  *N* (%) |
| --- | --- |
| Combined immunodeficiencies | 163 (100) |
| CIDs with associated or syndromic features | 98 (97.0) |
| Predominantly antibody deficiencies | 155 (100) |
| Diseases of immune dysregulation | 46 (93.9) |
| Congenital defects of phagocyte number or function | 17 (85.0) |
| Defects in intrinsic and innate immunity | 8 (66.7) |
| Complement deficiencies | 13 (8.5) |
| Other IEI | 14 (82.3) |
| Total | 514 (76.7) |

***Abbreviations:*** *CID, combined immunodeficiency; IEI, inborn errors of immunity; IUIS, international union of immunological societies.*
